# Cladodionen, a Cytotoxic Hybrid Polyketide from the Marine-Derived *Cladosporium* sp. OUCMDZ-1635

**DOI:** 10.3390/md16020071

**Published:** 2018-02-22

**Authors:** Guoliang Zhu, Fandong Kong, Yi Wang, Peng Fu, Weiming Zhu

**Affiliations:** 1 Key Laboratory of Marine Drugs, Ministry of Education of China, School of Medicine and Pharmacy, Ocean University of China, Qingdao 266003, China; guoliangzhu2015@hotmail.com (G.Z.); kongfandong501@126.com (F.K.); wangyi0213@ouc.edu.cn (Y.W.); 2 Institute of Tropical Bioscience and Biotechnology, Chinese Academy of Tropical Agricultural Sciences, Haikou 571101, China; 3 Laboratory for Marine Drugs and Bioproducts of Qingdao National Laboratory for Marine Science and Technology, Qingdao 266003, China

**Keywords:** marine-derived fungus, *Cladosporium* sp. OUCMDZ-1635, polyketide, cladodionen, cladosacid, cytotoxicities

## Abstract

A new hybrid polyketide, cladodionen (**1**), together with a new abscisic acid analogue, cladosacid (**2**), were isolated from the marine-derived fungus, *Cladosporium* sp. OUCMDZ-1635. Their structures, including the absolute configurations, were fully elucidated on the basis of spectroscopic analysis, ECD spectra, quantum chemical calculations, and chemical methods. Cladodionen (**1**) showed cytotoxic activities against MCF-7, HeLa, HCT-116, and HL-60 human cancer cell lines with IC_50_ values of 18.7, 19.1, 17.9, and 9.1 µM.

## 1. Introduction

**Figure 1:**
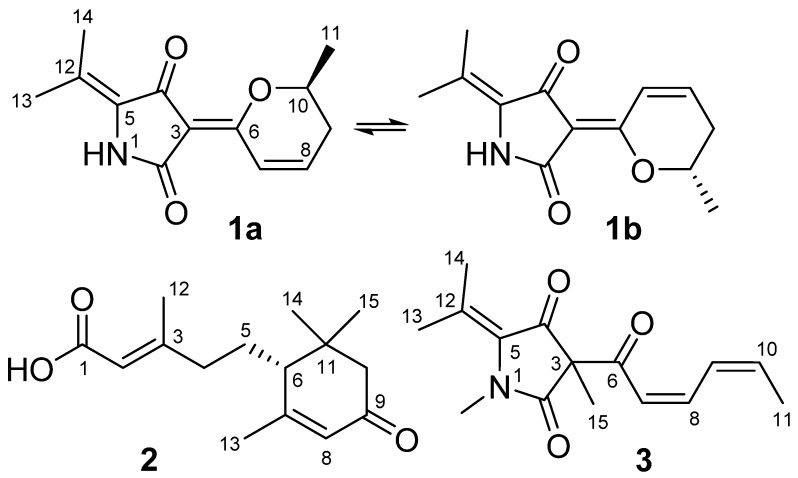
Chemical structures of compounds **1**–**3**.

The past decades have witnessed a huge development in the research on natural products from marine organisms, especially from marine microbes [1,2,3,4]. Due to their diverse structures and fascinating activities, metabolites from marine-derived fungi have attracted the attention of chemists and biologists [5,6,7]. According to our statistics, nearly two thousand compounds have been isolated from marine-derived fungi since 1945, many of which exhibit potent biological activities [8,9]. During the last decade, we have chemically investigated marine-derived fungal species isolated from many different marine habitats and discovered a variety of structurally interesting natural products with various biological activities, such as sclerotides A and B [10], cottoquinazolines B–D [11], penipenes A–F [12], and phomazines A–C [13]. As part of our studies to search for new bioactive compounds from marine-derived fungi, our attention was drawn to an endozoic fungal strain, *Cladosporium* sp. OUCMDZ-1635, isolated from a sponge (Figure S26). The ethyl acetate (EtOAc) extract of the fermentation broth was found to show moderate cytotoxicity against cancer cell lines at 100 μg/mL. Furthermore, HPLC-UV analysis of the EtOAc extract revealed one main peak with UV absorptions at 278 nm and 322 nm. Chemical investigation on this strain has resulted in the isolation of a new hybrid polyketide, cladodionen (**1**), and a new analogue of abscisic acid, which we named cladosacid (**2**). Cladodionen (**1**) was isolated as an inseparable mixture of two geometric isomers because of dynamic interconversion (Figure 1). Compound **1** represents a new natural skeleton and structurally belonged to hybrid polyketides, and only four examples, bripiodionen [14], apiodionen [15], vermelhotin [16] and hypoxyvermelhotins [17], have the similar skeleton (Figure S1). Hybrid polyketides are a kind of polyketide-amino acid hybrid fungal metabolites that normally arose from the tetramic acid derivatives and displayed diverse bioactivities [18].

## 2. Results and Discussion

### 2.1. Structure Elucidation

**Figure 2:**
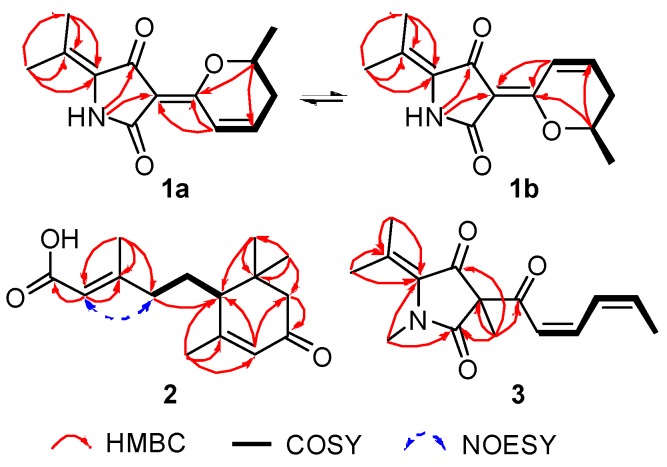
Key 2D NMR correlations for the structural assignment of **1**–**3**.

Cladodionen (**1**) was isolated as a brown powder and presented as an inseparable mixture of two *E*/*Z* geometric isomers (**1a** and **1b**). Although HPLC analysis of **1** through an ODS column (60% MeOH/H_2_O) showed that it afforded a baseline separation of the two isomers, attempts to separate the two isomers failed due to spontaneous and rapid isomerization between two forms. The positive ESI peak at *m*/*z* 234 [M + H]^+^ (Figure S8) and the HRESIMS peak at *m*/*z* 234.1131 [M + H]^+^ (Figure S9) indicated that the two isomers have the same molecular formula of C_13_H_15_NO_3_, requiring seven degrees of unsaturation. The ^1^H and ^13^C NMR spectra of **1** showed two sets of resonances with a ratio of 5:3 for the **1a** and **1b** isomers (Figures S2 and S3). The ^13^C NMR spectrum showed 13 carbon signals for each isomer that were classified by DEPT (Figure S4) and HMQC (Figure S5) as three methyl carbons, one sp^3^-methylene carbon, two sp^2^-methine carbons and one oxygenated sp^3^-methine carbon, and six non-protonated sp^2^-carbons (Table 1). The ^1^H NMR spectrum of **1a** showed six coupled signals at *δ*_H_ 1.39 (H_3_-11, d, *J =* 6.0 Hz), 4.41 (H-10, m), 2.36/2.56 (H-9, m), 6.93 (H-8, m), and 7.70 (H-7, d, *J =* 9.9 Hz). These data combined with ^1^H-^1^H COSY correlations extending from H-7 to H_3_-11 and the key HMBC correlations of H-7 to C-3/C-6 and H-10 to C-6/C-8 (Figure 2 and Figures S6 and S7) constituted the 5,6-dihydro-2*H*-pyran moiety. Two vinyl methyl groups at *δ*_H_ 1.74 (H-13, s) and 2.10 (H-14, s), along with the HMBC correlations of H_3_-13 to C-5/C-12/C-14, and H_3_-14 to C-5/C-12/C-13 (Figure 2 and Figure S7), indicated the presence of a 2-methylpropen-1-ylidene fragment. The relative positions of C-13 and C-14 was assigned from the lower filed proton signal of H_3_-14 (*δ*_H_ 2.10) than H_3_-13 (*δ*_H_ 1.74) due to the deshielding effect of 4-carbonyl group. The remaining ^1^H and ^13^C NMR signals and the HMBC correlations of HN-1 (*δ*_H_ 9.79) to C-3 (*δ*_C_ 103.2), C-4 (*δ*_C_ 183.3) and C-5 (*δ*_C_ 129.3) (Figure 2 and Figure S7), along with the careful comparison with known bripiodionen [14], suggested a 2,4-pyrrolidinedione moiety. Thus, compound **1** had the same core as bripiodionen.

**Scheme 1:**
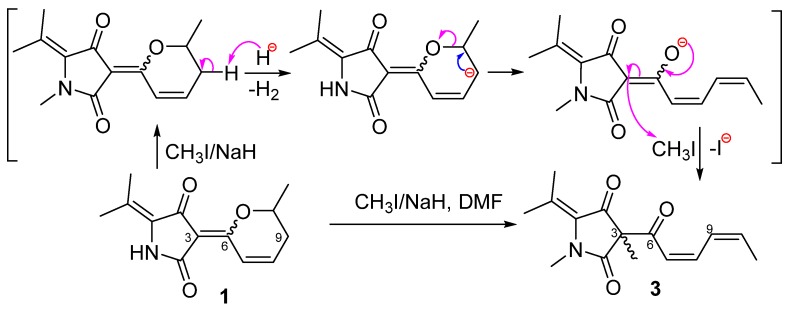
Methylation of cladodionen (**1**).

In order to confirm the constitution of **1**, a methylation was done by adding NaH and CH_3_I reagents to the *N*,*N*-dimethylformamide (DMF) solution of **1** (Scheme 1). The reaction product was purified and identified to be 3-((2*Z*,4*Z*)-hexa-2,4-dienoyl)-1,3-dimethyl-5-(propan-2-ylidene)pyrrolidine-2,4-dione (**3**) by MS (Figure S25), 1D and 2D NMR data (Figure 2 and Figures S19–S24). Compound **1** could undergo an *N*-methylation followed by an intramolecular elimination [19] and methylation under basic conditions to generate **3**. The postulated mechanism was showed in Scheme 1. Cladodionen (**1**) could undergo a nucleophilic reaction at *N*-1 with CH_3_I/NaH in DMSO solution to form *N*-methylcladodionen first followed by the deprotonation at C-9 of *N*-methylcladodionen to generate a carbanion intermediate. Then a cleavage of C–O sigma bond of the hydropyran ring took place to form the key intermediate, an enolate ion with an open chain, which underwent a nucleophilic substitution with CH_3_I to generate **3**.

**Figure 3:**
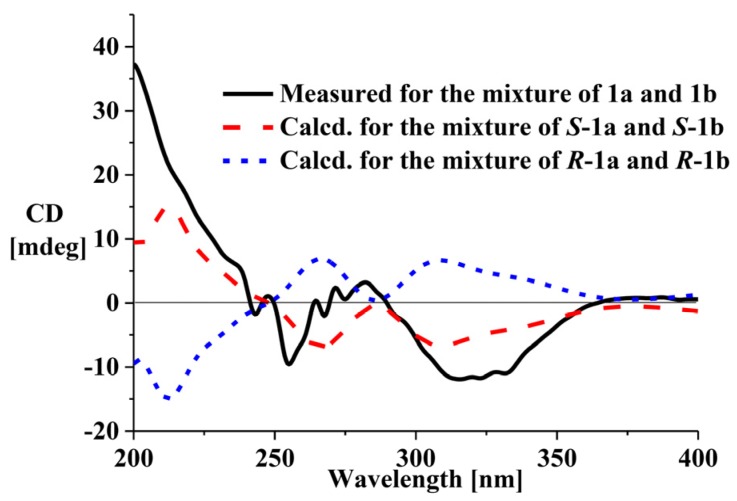
Measured and calculated ECD spectra for **1**.

The NMR data of **1a** and **1b** were nearly identical and the only significant differences between **1a** and **1b** were the chemical shifts of two carbonyls (*δ*_C-2_ 166.4/164.5, *δ*_C-4_ 183.3/185.8) (Table 1). These data indicated that **1a** and **1b** were a pair of rapidly interconverting *E*/*Z* geometric isomers in solution. Bripiodionen and apiodionen also displayed a similar equilibrium [14,15]. We carried out theoretical calculations of energy for stable conformers of **1a** and **1b**. The preliminary conformational distribution search was performed with the HyperChem software (Hyperchem Release 7.5, Hypercube, Inc., Gainesville, FL, USA). The corresponding minimum geometries were further fully optimized by using DFT at the B3LYP/6-31G(d) level as implemented in the Gaussian 03 program package (Gaussian 03 Revision E.01. Gaussian, Inc., Wallingford, CT, USA) [20]. The results (Table S1) showed that the stable conformers (**1a**-1, **1a**-2) of 1a possessed lower molecular energy than those of **1b** (**1b**-1, **1b**-2), and **1a** and **1b** accounted for 70.3% and 29.7% equilibrium populations, respectively, which was very close to their population in DMSO-*d_6_* solution. These data confirmed the *E*-Δ^3(6)^ and *Z*-Δ^3(6)^ for **1a** and **1b**, respectively. To establish the absolute configuration of **1a** and **1b**, electrostatic circular dichroism (ECD) spectra for the mixture of *R*-**1a** and *R*-**1b**, *S*-**1a** and *S*-**1b** were computed using the stable conformers obtained by the time-dependent density functional theory (TDDFT) [B3LYP/6-31G(d)] method [13], respectively. The measured ECD spectrum of the mixture matched well with calculated ECD spectrum for the mixture of *S*-**1a** and *S*-**1b**, indicating that the absolute configurations of **1a** and **1b** were both 10*S* (Figure 3).

**Figure 4:**
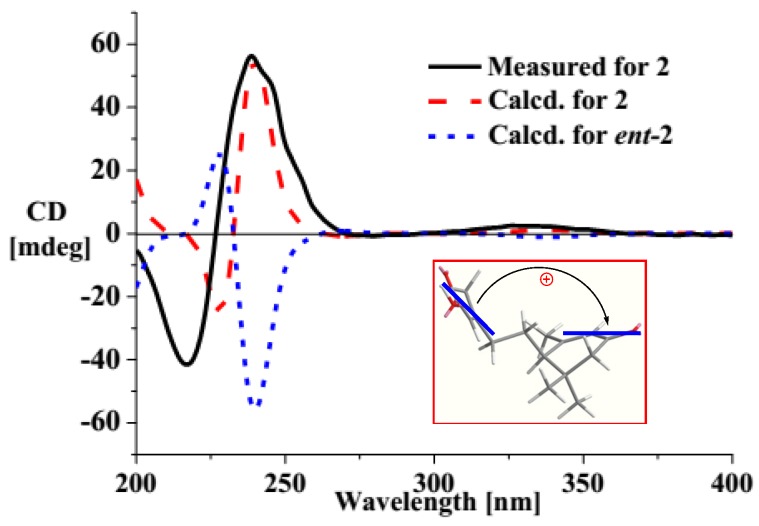
Measured and calculated ECD spectra and ECD exciton chirality model for **2**.

Cladosacid (**2**) was obtained as an amorphous powder and its molecular formula was determined to be C_15_H_20_O_3_ on the basis of a HRESIMS peak at *m*/*z* 249.1496 [M − H]^−^ (Figure S17) and *m*/*z* 251.1645 [M + H]^+^ (Figure S18). By means of DEPTQ and HSQC experiments (Figures S11 and S12), the 1D NMR spectra (Figures S10 and S11) displayed four methyls, three sp^3^ methylenes, two sp^2^ methines, one sp^3^ methine, and five non-protonated carbons including one sp^3^ carbon, two sp^2^ carbons, one carboxylic carbon and one conjugated ketone carbon (Table 1). The existence of a carboxylic group was suggested from the negative ion ESIMS at *m*/*z* 249 (Figure S16) as well as the very close *δ*_C-1_ value (170.4 ppm) to *δ*_COOH_ of callilongisin D (170.5 ppm) [21]. The key HMBC correlations of H_3_-14/H_3_-15 to C-6/C-10/C-11, H_3_-14 to C-15, H_2_-10 to C-6/C-8/C-9, H-8 to C-6/C-10, and H_3_-13 to C-6/C-7/C-8 (Figure 2 and Figure S14) revealed the presence of a 3,5,5-trimethyl-2-cyclohexenone nucleus. HMBC correlations of H-2 to C-1/C-3, and H_3_-12 to C-2/C-3/C-4 (Figure 2), along with the ^1^H-^1^H COSY correlation of H_2_-4 and H_2_-5 (Figure S13), suggested the presence of 4-carboxy-3-methylbut-3-en-1-yl group. Furthermore, the ^1^H-^1^H COSY correlation between H_2_-5 and H-6 located the substituent group at C-6 of the cyclohexenone nucleus. The NOESY correlation between H-2 and H-4 suggested *E*-geometry of Δ^2^-double bond (Figure S15). Both compound **2** and the known compound 9,10-dihydroxy-4-megastigmen-3-one [22] had the same cyclohexenone nucleus with a chiral C-6 carbon. The only difference between them was that the 4-carboxy-3-methylbut-3-en-1-yl group in **2** replaced the corresponding 3,4-dihydroxybutyl in 9,10-dihydroxy-4-megastigmen-3-one. Compound **2** and the 9,10-dihydroxy-4-megastigmen-3-one had similar UV (245 vs. 240 nm) but distinct ECD [239 (+0.44) and 218 (−0.32) vs. 350 (+0.21) and 274 (+0.81) nm] data, indicating that the α,β-unsaturated carboxylic acid and α,β-unsaturated ketone chromophores in **2** must interact with each other to generate exciton ECDs [23]. This phenomenon had also been found in the similar compound, (+)-trans-abscisic acid [24]. The ECD spectrum of compound **2** showed a positive Cotton effect, indicating the 6*R*- absolute configuration (Figure 4). Furthermore, ECD calculations of **2** and *ent*-**2** using the TDDFT method at the B3LYP/6-31G(d) level was performed [13]. The results showed that the measured ECD curve matched well with the calculated ECD of **2** and opposite to that of *ent*-**2** (Figure 4), further confirming 6*R*- configuration.

### 2.2. The Bioactivities of Compounds ***1**–**3*** from Cladosporium sp. OUCMDZ-1635.

Compounds **1** and **2** were tested for cytotoxic activities against human lung carcinoma cell line (A549), human breast adenocarcinoma cell line (MCF-7), human epithelioid cervix carcinoma cell line (HeLa), human colorectal carcinoma cell line (HCT-116), human erythromyeloblastoid leukemia cell line (K562) and human promyelocytic leukemia cell line (HL-60) using adriamycin (ADR) as the positive control. Only compound **1** showed cytotoxic activities against MCF-7, HeLa, HCT-116, and HL-60 cell lines with IC_50_ values of 18.7, 19.1, 17.9, and 9.1 µM, while IC_50_ values of ADR were 0.67, 0.32, 0.21 and 0.02 µM, respectively. However, compounds **1** and **2** did not show antibacterial activities against *Bacillus subtilis* CGMCC 1.3376, *Clostridium perfringens* CGMCC 1.0876, *Escherichia coli* ATCC 11775, *Pseudomonas aeruginosa* ATCC10145, *Staphylococcus aureus* ATCC 6538, and *Candida albicans* ATCC 10231 when tested at concentrations of 100 μg/mL.

## 3. Experimental Section

### 3.1. General Experimental Procedures

Optical rotations were measured on a JASCO P-1020 digital polarimeter, and UV spectra were recorded on a Beckman DU 640 spectrophotometer. ECD data were collected using a JASCO J-715 spectropolarimeter. IR spectra were obtained on a Nicolet Nexus 470 spectrophotometer as KBr discs. NMR spectra were recorded using a JEOL JNM-ECP 600 spectrometer or an Agilent 500 MHz DD2 spectrometer using TMS as an internal standard or residual solvent signals for referencing. HRESIMS spectra were determined using a Micromass^®^ Q-TOF Ultima Global GAA076 LC mass spectrometer (Waters Aisa, Ltd. Singapore). Semi-preparative HPLC was carried out using an ODS column (YMC-pack ODS-A, 10 × 250 mm, 5 μm, 4 mL/min). Thin layer chromatography (TLC) and column chromatography (CC) were performed on plates pre-coated with silica gel GF254 (10–40 μm, Qingdao Marine Chemical Factory, Qingdao, China) and Sephadex LH-20 (Amersham Biosciences, Buckinghamshire, England), respectively. Vacuum-liquid chromatography (VLC) utilized silica gel H (Qingdao Marine Chemical Factory).

### 3.2. Collection and Phylogenetic Analysis of Strain OUCMDZ-1635

The fungus OUCMDZ-1635 was isolated from a sponge sample (Figure S26) collected from Xisha Islands of China in August 2010. After it was ground into powder, the sample (1.0 g) was diluted to 10^−2^ g/mL with sterile water, 100 μL of which was deposited on a PDA (200 g potato, 20 g glucose, 20 g agar per liter of sea water) plate containing chloramphenicol (100 μg/mL) as a bacterial inhibitor. A single colony was transferred onto another PDA plate and was identified as *Cladosporium* sp. according to its morphological characteristics and ITS gene sequences (GenBank accession No. KT336457). A reference culture of OUCMDZ-1635 is maintained at −80 °C and deposited in W. Zhu’s laboratory.

### 3.3. Cultivation and Extraction of OUCMDZ-1635

Fungal strain OUCMDZ-1635 was cultured on slants of PDA medium at 28 °C for 5 days. Plugs of agar supporting mycelium growth were cut and transferred aseptically to 20 × 1000 mL Erlenmeyer flasks each containing rice medium composed of 80 g rice and 120 mL seawater. The flask was incubated at room temperature under static conditions for 30 days. The cultures were extracted three times by EtOAc (each 300 mL) and the combined EtOAc extracts were dried in vacuo to yield 6.5 g of extract.

### 3.4. Purification

The extract (6.5 g) was fractionated by a silica gel VLC column, eluting with a step gradient of MeOH and CH_2_Cl_2_ (0–100%), and 20 fractions (Fr.1–Fr.20) were collected. Fraction 4 (0.8 g) was subjected to Sephadex LH-20 chromatography (10 × 400 cm) with CH_2_Cl_2_/MeOH (1:1) to afford three subfractions (Fr.4.1–Fr.4.3). Fr.4.3 (87 mg) was further purified by HPLC on an ODS column (60% MeOH/H_2_O) to give compound **1** (30.0 mg, t_R_ = 6.5 min). Fr.4.2 (43 mg) was purified by HPLC on an ODS column (60% MeOH/H_2_O) to yield compound **2** (2.0 mg, t_R_ = 12.6 min).

**Cladodionen (1)**: brown powder; $\alpha_{D}^{25}$ −10.8 (c 0.3, MeOH); UV (MeOH) λ_max_ (log ε) 278 (3.43), 322 (4.04) nm; ECD (0.002 *M*, MeOH) λ_max_ (Δε) 255 (−0.15), 318 (−0.17); IR (KBr) ν_max_ 3385, 2875, 1719, 1680, 1458, 1205 cm^−1^; ^1^H and ^13^C NMR data, see Table 1; HRESIMS *m*/*z* 234.1131 [M + H]^+^ (calcd. for C_13_H_16_NO_3_, 234.1125); ESIMS *m*/*z* 234 [M + H]^+^.

**Cladosacid (2)**: amorphous powder; $\alpha_{D}^{25}$ + 41.8 (c 0.1, MeOH); UV (MeOH) λ_max_ (log ε) 245 (3.11) nm; ECD (0.004 *M*, MeOH) λ_max_ (Δε) 239 (+0.44), 218 (−0.32); IR (KBr) ν_max_ 3478, 2929, 1766, 1384, 1096 cm^−1^; ^1^H and ^13^C NMR data, see Table 1; HRESIMS *m*/*z* 251.1645 [M + H] ^+^ (calcd. for C_15_H_23_O_3_, 251.1642) and *m*/*z* 249.1496 [M − H]^−^ (calcd. for C_15_H_21_O_3_, 249.1496); ESIMS *m*/*z* 249 [M − H]^−^.

### 3.5. Preparation of **3**

To a stirred suspension of sodium hydride (6.0 mg, mixture of 60% NaH in mineral oil) in *N*,*N*- dimethylformamide (DMF) (1.0 mL) was added a solution of compound **1** (5.0 mg) in DMF (1.0 mL) at −5 °C. After stirring for 0.5 h, 15 μL of CH_3_I was added dropwise and the mixture was stirred for another 0.5 h. Then the reaction was quenched by the addition of saturated aqueous NH_4_Cl solution (2.0 mL) and extracted with CH_2_Cl_2_ (3 × 10 mL). The organic layers were combined and washed with H_2_O (2 × 10 mL) and concentrated in vacuum. The residue was then purified by HPLC over an ODS column (70% MeOH/H_2_O) to afford compound **3** (4.5 mg, t_R_ = 6.45 min, 80% yield).

**3-((2*Z*,4*Z*)-Hexa-2,4-dienoyl)-1,3-dimethyl-5-(propan-2-ylidene)pyrrolidine-2,4-dione (3)**: a yellow powder; $\alpha_{D}^{25}$ −5.6 (c 0.1, MeOH); ^1^H and ^13^C NMR data, see Table 1; ESIMS *m*/*z* 284 [M + Na]^+^.

## 4. Conclusions

A new hybrid polyketide with a 3-(2*H*-pyran-2-ylidene)pyrrolidine-2,4-dione nucleus, cladodionen (**1**), along with a new abscisic acid analogue, cladosacid (**2**), were isolated and identified from a solid cultural products of the marine-derived *Cladosporium* sp. OUCMDZ-1635. Cladodionen (**1**) exhibit cytotoxic activities against MCF-7, HeLa, HCT-116, and HL-60 human cancer cell lines with the IC_50_ values of 18.7, 19.1, 17.9, and 9.1 µM.

## Figures and Tables

**Table 1:** ^1^H and ^13^C NMR Data for Compounds **1**–**3**.

No.	1a *^a^*		1b *^a^*		2 *^b^*		3 *^c^*	
	*δ*_C_	*δ*_H_, Mult. (*J* in Hz)	*δ*_C_	*δ*_H_, Mult. (*J* in Hz)	*δ*_C_	*δ*_H_, Mult. (*J* in Hz)	*δ*_C_	*δ*_H_, Mult. (*J* in Hz)
1		9.79, s	103.1, C	9.64, s	161.9, C		65.5, C	
2	166.4, C		164.5, C		115.5, CH	5.70, s	170.0, C	
3	103.2, C		103.1, C		161.9, C			
4	183.3, C		185.8, C		40.9, CH_2_	2.26, t (8.0)	194.2, C	
5	129.3, C		130.0, C		28.2, CH_2_	1.58, m; 1.89*^d^*	131.9, C	
6	165.9, C		167.5, C		50.8, CH	1.89*^d^*	188.7, C	
7	119.9, CH	7.70, d (9.9)	119.9, CH	7.67, d (9.8)	164.6, C		117.6, CH	5.77, d (11.3)
8	143.5, CH	6.93, m	144.1, CH	6.96, m	125.6, CH	5.85, s	147.3, CH	6.66, dd (11.3, 10.7)
9	30.4, CH_2_	2.36, m; 2.56, m	30.4, CH	2.34, m; 2.59, m	199.2, C		128.7, CH	7.22, dd (10.7, 10.3)
10	73.4, CH	4.41, m	73.4, CH	4.41, m	47.3, CH_2_	2.06, d (17.1); 2.37, d (17.1)	145.5, CH	6.34, dq (10.3, 6.8)
11	20.1, CH_3_	1.39, d (6.0)	20.1, CH_3_	1.39, d (6.0)	36.5, C		18.8, CH_3_	1.85, d (6.8)
12	118.4, C		118.8, C		19.3, CH_3_	2.18, s	127.7, C	
13	20.8, CH_3_	1.74, s	20.9, CH_3_	1.74, s	24.8, CH_3_	1.99, s	21.8, CH_3_	2.13, s
14	18.2, CH_3_	2.10, s	17.9, CH_3_	2.11, s	27.3, CH_3_	1.08, s	21.4, CH_3_	2.19, s
15					28.9, CH_3_	1.03, s	15.3, CH_3_	1.30, s

*^a^* Data were measured in DMSO-*d*_6_ at 600 MHz (^1^H) and 150 MHz (^13^C). *^b^* Data were measured in CDCl_3_ at 500 MHz (^1^H) and 125 MHz (^13^C). *^c^* Data were measured in DMSO-*d*_6_ at 500 MHz (^1^H) and 125 MHz (^13^C), and the signals for *N*-methyl were *δ* 3.36 (s, 3H) and 31.1 (CH_3_), respectively. *^d^* overlapped.

## Supplementary Materials

The following are available online at http://www.mdpi.com/1660-3397/16/2/71/s1, Bioassay protocols, Theory and calculation details, Table S1: Stable conformers of compounds **1a** and **1b**, Table S2: Stable conformers of compound **2**, Figure S1: Structures of bripiodionen, apiodionen, vermelhotin and hypoxyvermelhotins, Figure S2: ^1^H-NMR (600 MHz) spectrum of cladodionen (**1**) in DMSO-*d*_6_, Figure S3: ^13^C-NMR (150 MHz) spectrum of cladodionen (**1**) in DMSO-*d*_6_, Figure S4: DEPT (150 MHz) spectrum of cladodionen (**1**) in DMSO-*d*_6_, Figure S5: HMQC (600 MHz) spectrum of cladodionen (**1**) in DMSO-*d*_6_, Figure S6: ^1^H-^1^H COSY (600 MHz) spectrum of cladodionen (**1**) in DMSO-*d*_6_, Figure S7: HMBC (600 MHz) spectrum spectrum of cladodionen (**1**) in DMSO-*d*_6_, Figure S8: Positive ESIMS spectrum of cladodionen (**1**), Figure S9: Positive HRESIMS spectrum of cladodionen (**1**), Figure S10: ^1^H-NMR (500 MHz) spectrum of cladosacid (**2**) in CDCl_3_, Figure S11: ^13^C-DEPTQ (125 MHz) spectrum of cladosacid (**2**) in CDCl_3_, Figure S12: HSQC (500 MHz) spectrum of cladosacid (**2**) in CDCl_3_, Figure S13: ^1^H-^1^H COSY (500 MHz) spectrum of cladosacid (**2**) in CDCl_3_, Figure S14: HMBC (500 MHz) spectrum of cladosacid (**2**) in CDCl_3_, Figure S15: NOESY (500 MHz) spectrum of cladosacid (**2**) in CDCl_3_, Figure S16: Negative ESIMS spectrum of cladosacid (**2**), Figure S17: Negative HRESIMS spectrum of cladosacid (**2**), Figure S18: Positive HRESIMS spectrum of cladosacid (**2**), Figure S19: ^1^H-NMR (500 MHz) spectrum of compound **3** in DMSO-*d*_6_, Figure S20: ^13^C-NMR (125 MHz) spectrum of compound **3** in DMSO-*d*_6_, Figure S21: DEPT (125 MHz) spectrum of compound **3** in DMSO-*d*_6_, Figure S22: HMQC (500 MHz) spectrum of compound **3** in DMSO-*d*_6_, Figure S23: ^1^H-^1^H COSY (500 MHz) spectrum of compound **3** in DMSO-*d*_6_, Figure S24: HMBC (500 MHz) spectrum of compound **3** in DMSO-*d*_6_, Figure S25: Positive ESIMS spectrum of compound **3**, Figure S26: The ecological picture of the sponge sample.
