# Supplementary material for: Cladodionen, a Cytotoxic Hybrid Polyketide from the Marine-Derived Cladosporium sp. OUCMDZ-1635
Source: Mar Drugs. 2018 Feb 22;16(2):71. doi: 10.3390/md16020071 (PMC5852499; doi:10.3390/md16020071)

Cladodionen, a Cytotoxic Hybrid Polyketide from the Marine-Derived *Cladosporium* sp. OUCMDZ-1635

Guoliang Zhu [1,3], Fandong Kong[1,3,4], Yi Wang[1], Peng Fu[1,*] and Weiming Zhu[1,2,*]

1 Key Laboratory of Marine Drugs, Ministry of Education of China, School of Medicine and Pharmacy, Ocean University of China, Qingdao 266003, China; E-mails: guoliangzhu2015@hotmail.com (G. Zhu); kongfandong501@126.com (F. Kong); wangyi0213@ouc.edu.cn (Y. Wang); fupeng@ouc.edu.cn (P. Fu); weimingzhu@ouc.edu.cn (W. Zhu).

2 Laboratory for Marine Drugs and Bioproducts of Qingdao National Laboratory for Marine Science and Technology, Qingdao 266003, China.

3 These authors contributed equally to this paper.

4 F. Kong is now working in the Institute of Tropical Bioscience and Biotechnology, Chinese Academy of Tropical Agricultural Sciences, Haikou, 571101, China

* Author to whom correspondence should be addressed; E-Mail: fupeng@ouc.edu.cn (P. Fu); weimingzhu@ouc.edu.cn (W. Zhu); Tel/Fax: +86-532-82031268 (W. Zhu).

Table of contents

Bioassay Protocols........S2

Theory and Calculation Details........S3

Table S1. Stable conformers of compounds **1a** and **1b**........S3

Table S2. Stable conformers of compound **2**........S4

Figure S1. Structures of bripiodionen, apiodionen, vermelhotin and hypoxyvermelhotins........S5

Figure S2. ^{1}H-NMR (600 MHz) spectrum of cladodionen (**1**) in DMSO-d_6........S6

Figure S3. ^{13}C-NMR (150 MHz) spectrum of cladodionen (**1**) in DMSO-d_6........S7

Figure S4. DEPT (150 MHz) spectrum of cladodionen (**1**) in DMSO-d_6........S8

Figure S5. HMQC (600 MHz) spectrum of cladodionen (**1**) in DMSO-d_6........S9

Figure S6. ^{1}H-^{1}H COSY (600 MHz) spectrum of cladodionen (**1**) in DMSO-d_6........S10

Figure S7. HMBC (600 MHz) spectrum spectrum of cladodionen (**1**) in DMSO-d_6........S11

Figure S8. Positive ESIMS spectrum of cladodionen (**1**)........S12

Figure S9. Positive HRESIMS spectrum of cladodionen (**1**)........S13

Figure S10. ^{1}H-NMR (500 MHz) spectrum of cladosacid (**2**) in $CDCl_3$........S14

Figure S11. ^{13}C-DEPTQ (125 MHz) spectrum of cladosacid (**2**) in $CDCl_3$........S15

Figure S12. HSQC (500 MHz) spectrum of cladosacid (**2**) in $CDCl_3$........S16

Figure S13. ^{1}H-^{1}H COSY (500 MHz) spectrum of cladosacid (**2**) in $CDCl_3$........S17

Figure S14. HMBC (500 MHz) spectrum of cladosacid (**2**) in $CDCl_3$........S18

Figure S15. NOESY (500 MHz) spectrum of cladosacid (**2**) in $CDCl_3$......S19
Figure S16. Negative ESIMS spectrum of cladosacid (**2**)......S20
Figure S17. Negative HRESIMS spectrum of cladosacid (**2**)......S21
Figure S18. Positive HRESIMS spectrum of cladosacid (**2**)......S22
Figure S19. ^{1}H-NMR (500 MHz) spectrum of compound **3** in DMSO-d_6......S23
Figure S20. ^{13}C-NMR (125 MHz) spectrum of compound **3** in DMSO-d_6......S24
Figure S21. DEPT (125 MHz) spectrum of compound **3** in DMSO-d_6......S25
Figure S22. HMQC (500 MHz) spectrum of compound **3** in DMSO-d_6......S26
Figure S23. ^{1}H-^{1}H COSY (500 MHz) spectrum of compound **3** in DMSO-d_6......S27
Figure S24. HMBC (500 MHz) spectrum of compound **3** in DMSO-d_6......S28
Figure S25. Positive ESIMS spectrum of compound **3**......S29
Figure S26. The ecological picture of the sponge sample......S30

Bioassay Protocols

Antibiotic assays. The antibiotic activities against *Bacillus subtilis* CGMCC 1.3376, *Escherichia coli* ATCC 11775, *Clostridium perfringens* CGMCC 1.0876, *Pseudomonas aeruginosa* ATCC10145, *Staphylococcus aureus* ATCC 6538, and *Candida albicans* ATCC 10231 were evaluated by an agar dilution method. The tested strains were cultivated in LB agar plates for bacteria and in YPD agar plates for *C. albicans* at 37 °C. Compounds **1**, **2** and positive controls (ciprofloxacin lactate for bacteria and ketoconazole for *C. albicans*) were dissolved in MeOH at the concentration of 100 μg/mL. A 10 μL quantity of test solution was absorbed by a paper disk (5 mm diameter) and placed on the assay plates. After 24 h incubation, zones of inhibition (mm in diameter) were recorded. If the inhibition zone was observed, the compounds diluted to different concentrations by the continuous 2-fold dilution methods. The minimum inhibitory concentrations (MICs) were defined as the lowest concentration at which no microbial growth could be observed.

Cytotoxic assays. Cytotoxicity was assayed by the MTT[S1] and CCK-8[S2] methods. In the MTT assay, A549, MCF-7, HeLa, HCT-116, and HL-60 cell lines were grown in RPMI-1640 supplemented with 10% FBS under a humidified atmosphere of 5% CO_2 and 95% air at 37 °C, respectively. Cell suspension, 100 μL, at a density of 3 × 10^4 cell /mL was plated in 96-well microtiter plates, allowed to attach overnight, and then exposed to varying concentrations (10^{-5}–10^{-12} M) of compounds for 72 h. The MTT solution (20 μL, 5 mg/mL in IPMI-1640 medium) was then added to each well and incubated for 4 h. Old medium containing MTT was then gently replaced by DMSO and pipetted to dissolve any formazan crystals formed. Absorbance was then determined on a Spectra Max Plus plate

reader at 570 nm. In the CCK-8 assay, K562 cell line was grown in RPMI-1640 supplemented with 10% FBS under a humidified atmosphere of 5% CO_2 and 95% air at 37 °C. Cell suspension, 100 μL, at a density of 5 × 10^5 cell mL^{-1} was plated in 96-well microtiter plates and then exposed to varying concentrations (10^{-5}–10^{-12} M) of compounds after cultivation for 24 h. Three days later, 10 μL of CCK-8 solution was added 4 h before detection. Then the absorbency (A450 value) was measured, and the growth rates of cells were computed.

Theory and Calculation Details. The calculations were performed by using the density functional theory (DFT) as carried out in the Gaussian 03.[S3] The preliminary conformational distributions search were performed by HyperChem 7.5 software. All ground-state geometries were optimized at the B3LYP/6-31G(d) level. Conformers within a 2 kcal/mol energy threshold from the global minimum were selected to calculate the electronic transitions.[S4] The overall theoretical ECD spectra were obtained according to the Boltzmann weighting of each conformers. The percentage of each conformer for the mixture of **1a** and **1b** was calculated from the energy combined with the ratio between two isomers. Solvent effects of methanol solution were evaluated at the same DFT level by using the SCRF/PCM method.[S5]

(S1) Mosmann, T. J. *Immunol. Methods* **1983**, *65*, 55–63.

(S2) Xiong, T.; Chen, X.; Wei, H.; Xiao, H. *Arch. Med. Sci.* **2015**, *11*, 301–306.

(S3) Gaussian 03, Revision E.01, M. J. Frisch, G. W. Trucks, H. B. Schlegel, G. E. Scuseria, M. A. Robb, J. R. Cheeseman, J. A. Montgomery, Jr., T. Vreven, K. N. Kudin, J. C. Burant, J. M. Millam, S. S. Iyengar, J. Tomasi, V. Barone, B. Mennucci, M. Cossi, G. Scalmani, N. Rega, G. A. Petersson, H. Nakatsuji, M. Hada, M. Ehara, K. Toyota, R. Fukuda, J. Hasegawa, M. Ishida, T. Nakajima, Y. Honda, O. Kitao, H. Nakai, M. Klene, X. Li, J. E. Knox, H. P. Hratchian, J. B. Cross, V. Bakken, C. Adamo, J. Jaramillo, R. Gomperts, R. E. Stratmann, O. Yazyev, A. J. Austin, R. Cammi, C. Pomelli, J. W. Ochterski, P. Y. Ayala, K. Morokuma, G. A. Voth, P. Salvador, J. J. Dannenberg, V. G. Zakrzewski, S. Dapprich, A. D. Daniels, M. C. Strain, O. Farkas, D. K. Malick, A. D. Rabuck, K. Raghavachari, J. B. Foresman, J. V. Ortiz, Q. Cui, A. G. Baboul, S. Clifford, J. Cioslowski, B. B. Stefanov, G. Liu, A. Liashenko, P. Piskorz, I. Komaromi, R. L. Martin, D. J. Fox, T. Keith, M. A. Al-Laham, C. Y. Peng, A. Nanayakkara, M. Challacombe, P. M. W. Gill, B. Johnson, W. Chen, M. W. Wong, C. Gonzalez, and J. A. Pople, Gaussian, Inc., Wallingford CT, 2004.

(S4) (a) Casida, M. E. In Recent Advances in Density Functional Methods, part I; Chong, D. P., Eds.; World Scientific: Singapore, 1995; pp 155–192. (b) Gross, E. K. U.; Dobson, J. F.; Petersilka, M. Top. *Curr. Chem.* **1996**, *181*, 81–172. (c) Gross, E. K. U.; Kohn, W. *Adv. Quantum Chem.* **1990**, *21*, 255–291. (d) Runge, E.; Gross, E. K. U. *Phys. Rev. Lett.* **1984**, *52*, 997–1000.

(S5) (a) Miertus, S.; Tomasi, J. *Chem. Phys.* **1982**, *65*, 239–245. (b) Tomasi, J.; Persico, M. *Chem. Rev.* **1994**, *94*, 2027–2094. (c) Cammi, R.; Tomasi, J. *J. Comp. Chem.* **1995**, *16*, 1449–1458.

Table S1. Stable conformers of **1a** and **1b**.

Conformer	Conformation	Energy (kcal/mol)	Percent (%)
**1a**-1	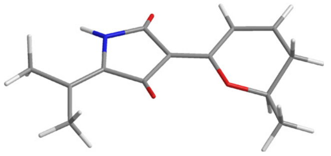	-492489.6333	65.61

**1a**-2	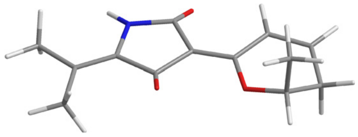	-492488.0722	4.69
**1b**-1	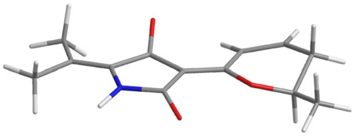	-492489.1136	27.74
**1b**-2	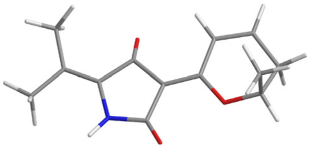	-492487.5573	1.96

Table S2. Stable conformers of compound **2**.

**Conformer**	**Conformation**	**Energy (kcal/mol)**	**Percent (%)**
**2**-1	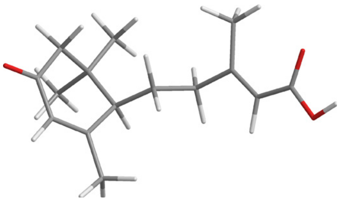	-508607.0081	20.9
**2**-2	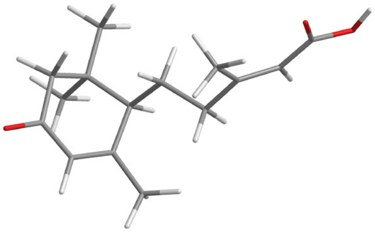	-508606.9121	17.77
**2**-3	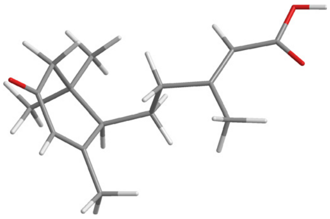	-508606.7897	14.45
**2**-4	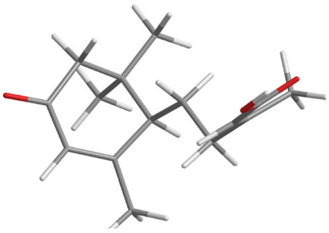	-508606.7851	14.34

**2**-5	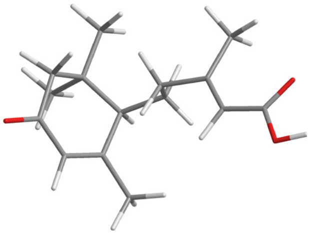	-508606.7639	13.84
**2**-6	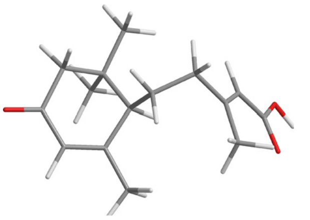	-508606.4885	8.69
**2**-7	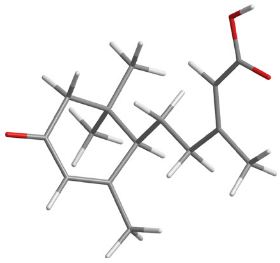	-508606.3384	6.74
**2**-8	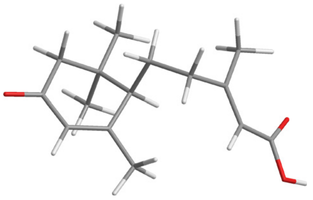	-508605.7806	2.63
**2**-9	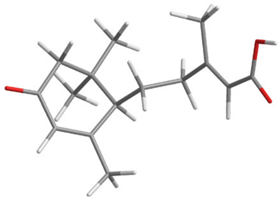	-508604.9466	0.64

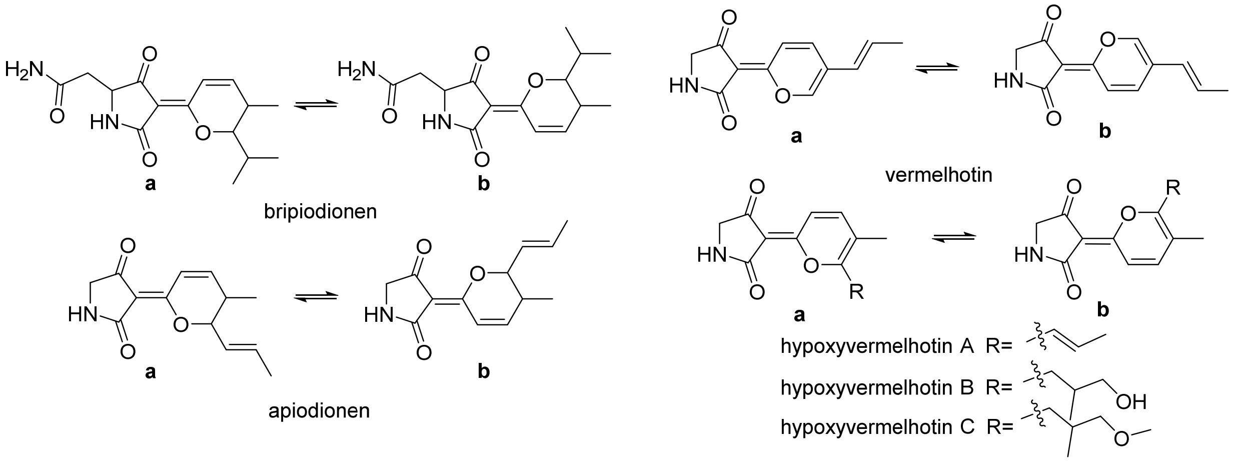

Figure S1. Structures of bripiodionen, apiodionen, vermelhotin and hypoxyvermelhotins.

Figure S2. ^{1}H-NMR (600 MHz) spectrum of cladodionen (**1**) in DMSO-d_6

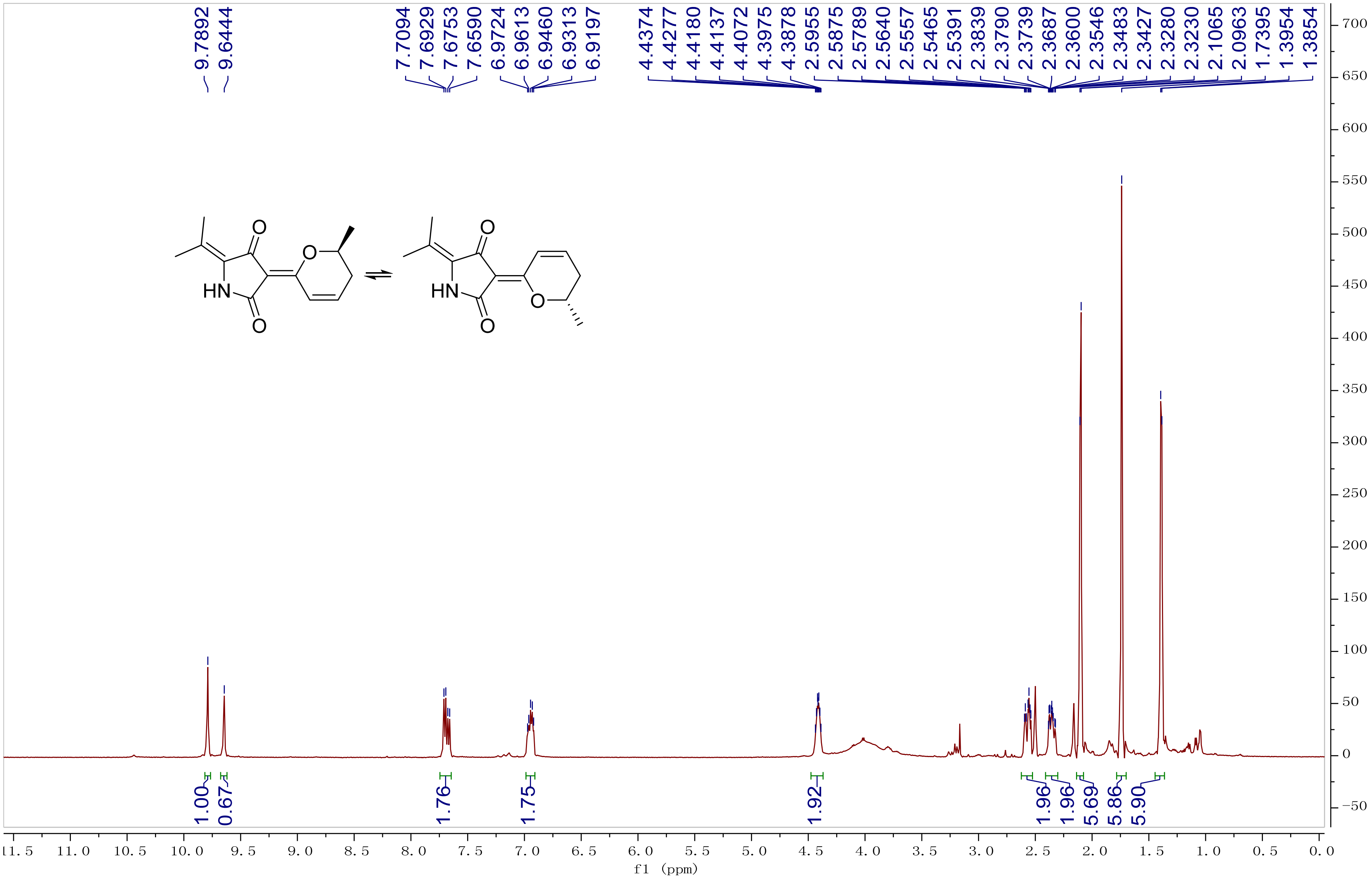

Figure S3. ^{13}C-NMR (150 MHz) spectrum of cladodionen (**1**) in DMSO-d_6

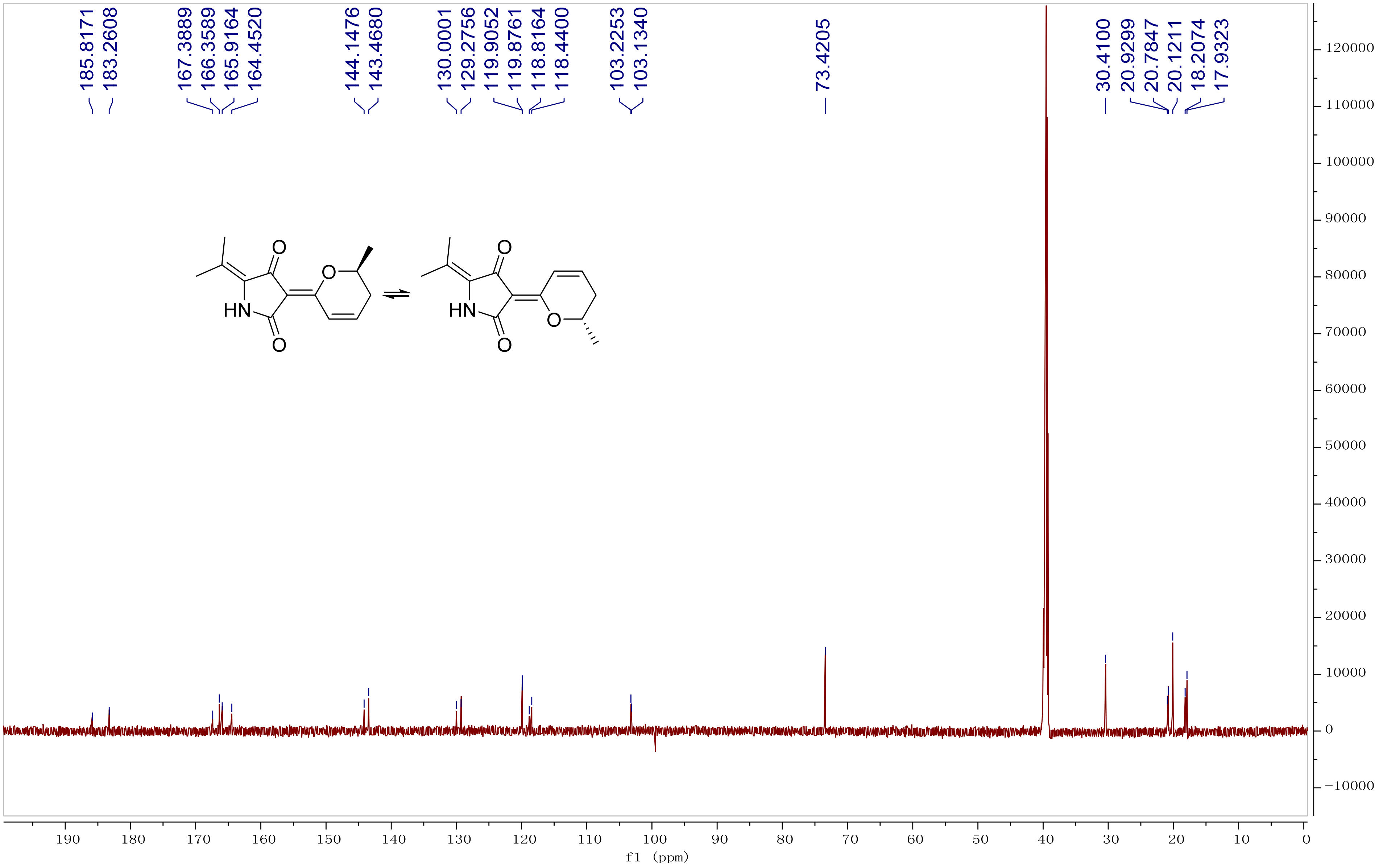

Figure S4. DEPT (150 MHz) spectrum of cladodionen (**1**) in DMSO-d_6

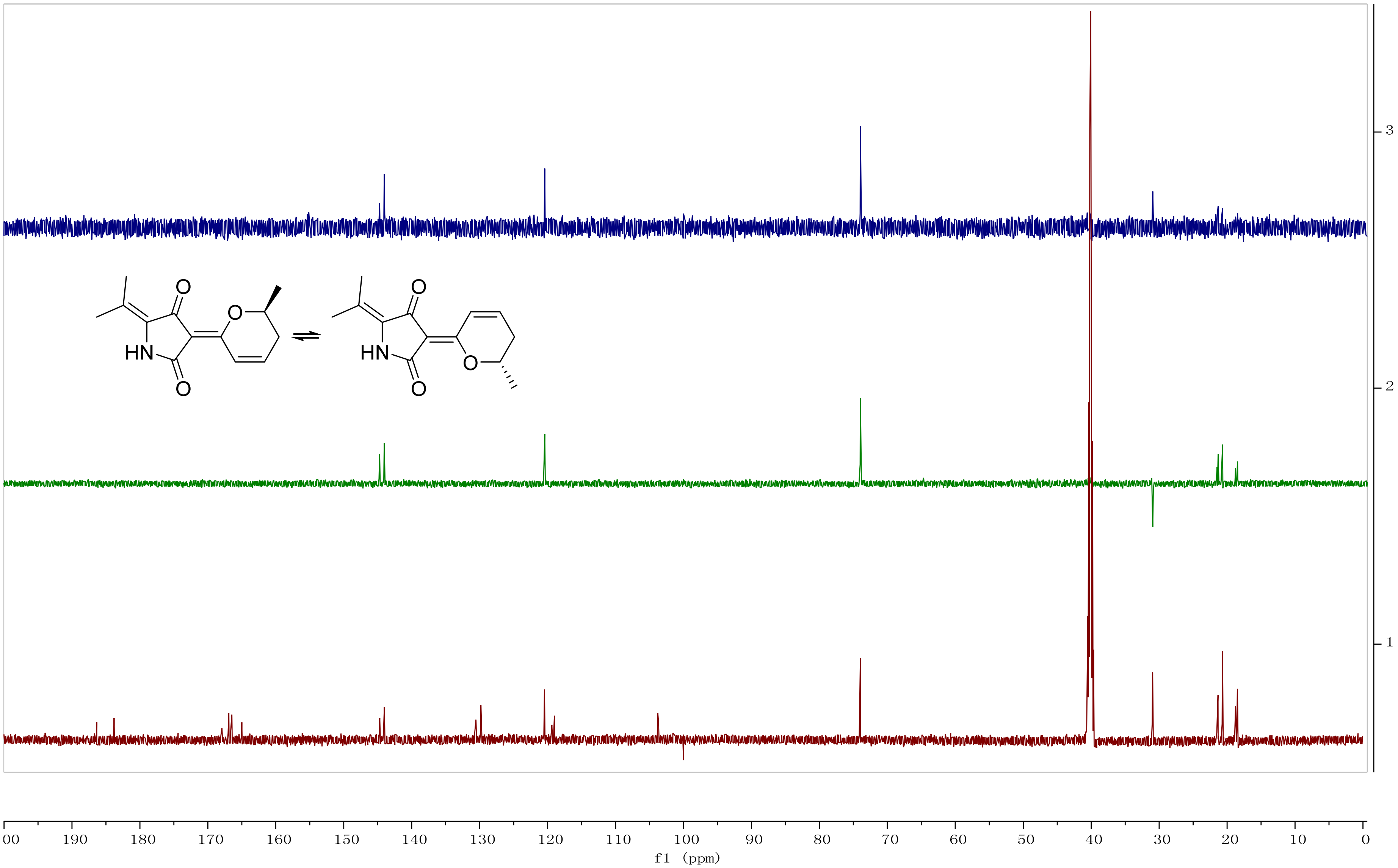

Figure S5. HMQC (600 MHz) spectrum of cladodionen (**1**) in DMSO-d_6

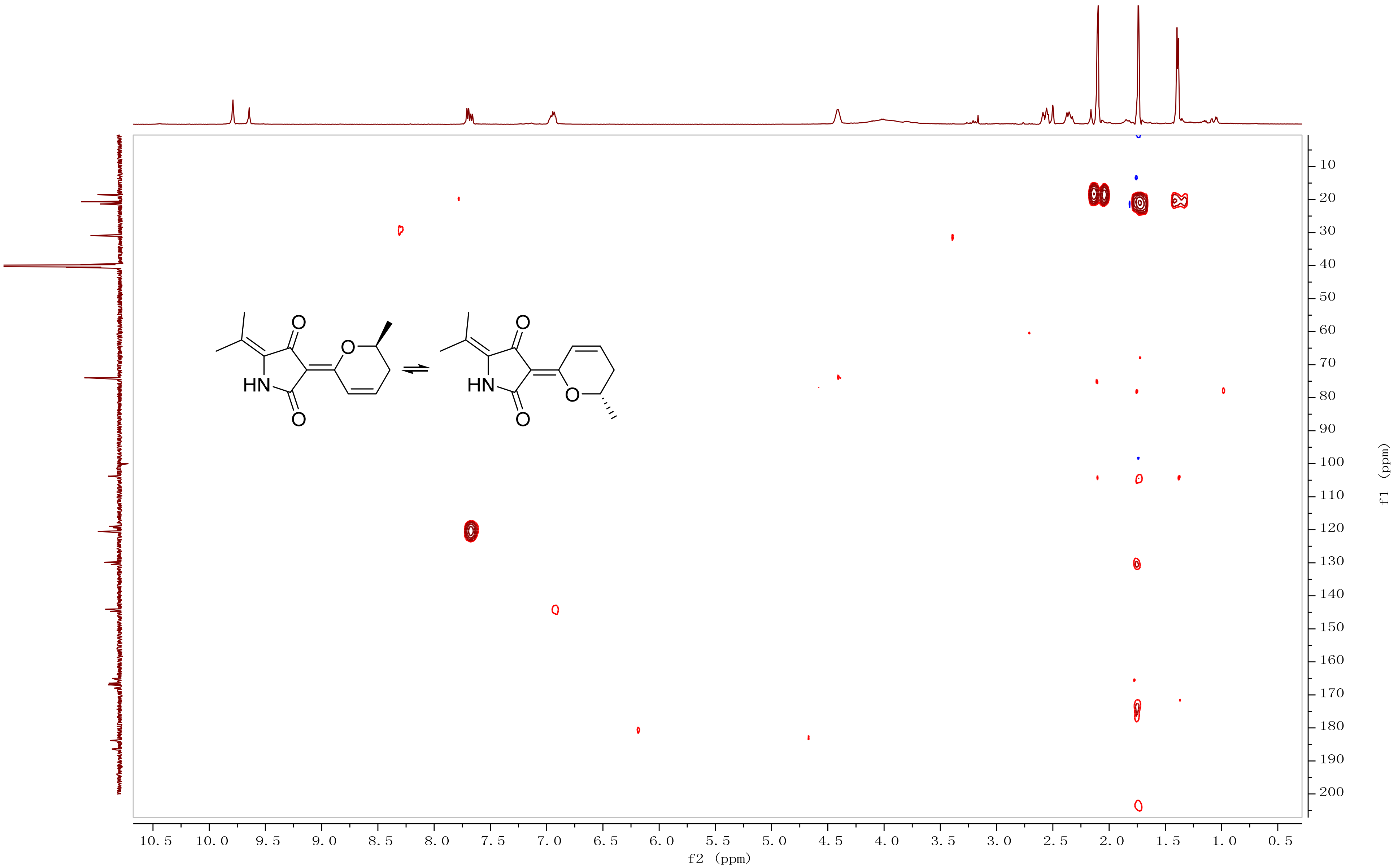

Figure S6. ^{1}H-^{1}H COSY (600 MHz) spectrum of cladodionen (**1**) in DMSO-d_6

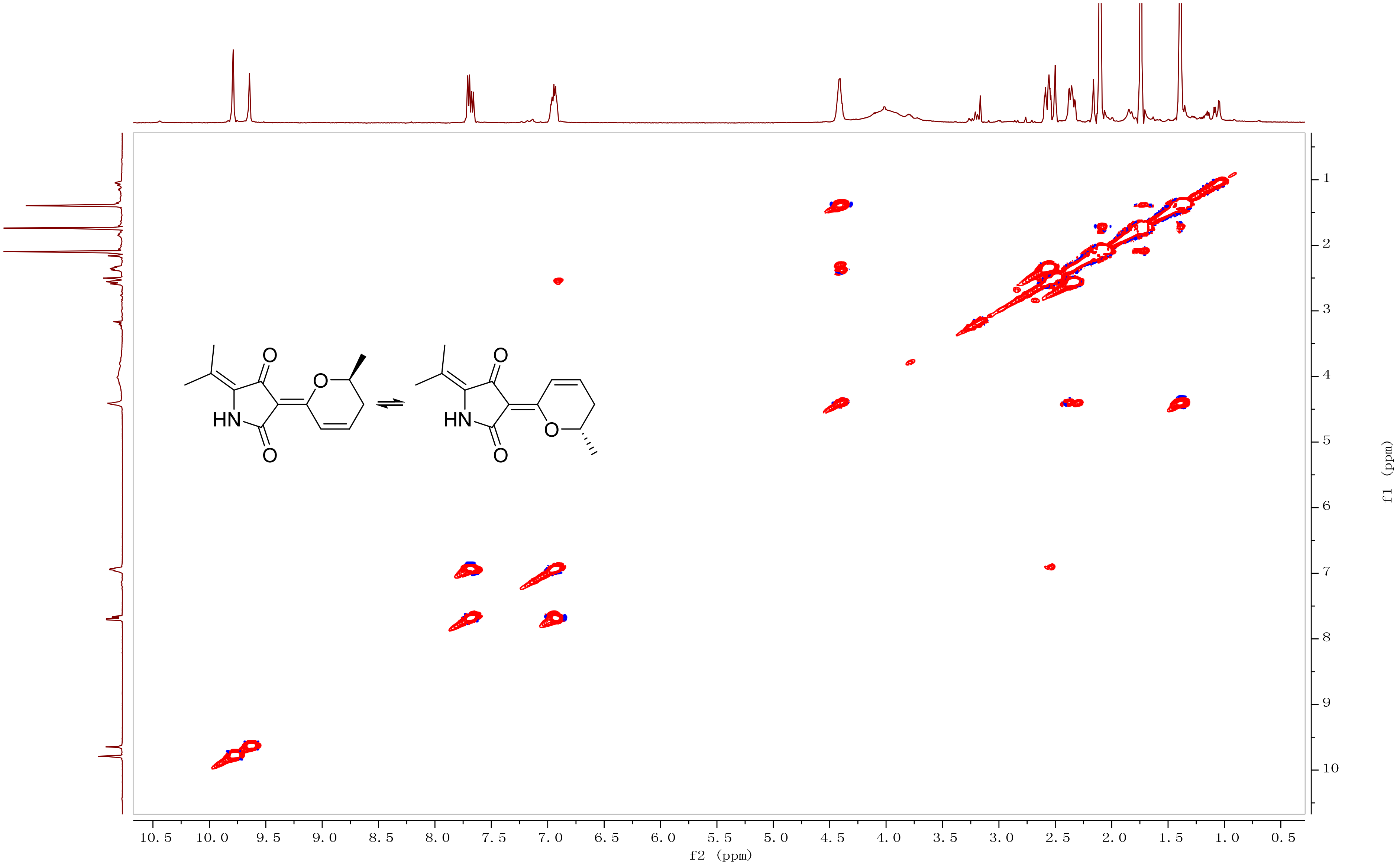

Figure S7. HMBC (600 MHz) spectrum of cladodionen (**1**) in DMSO-d_6

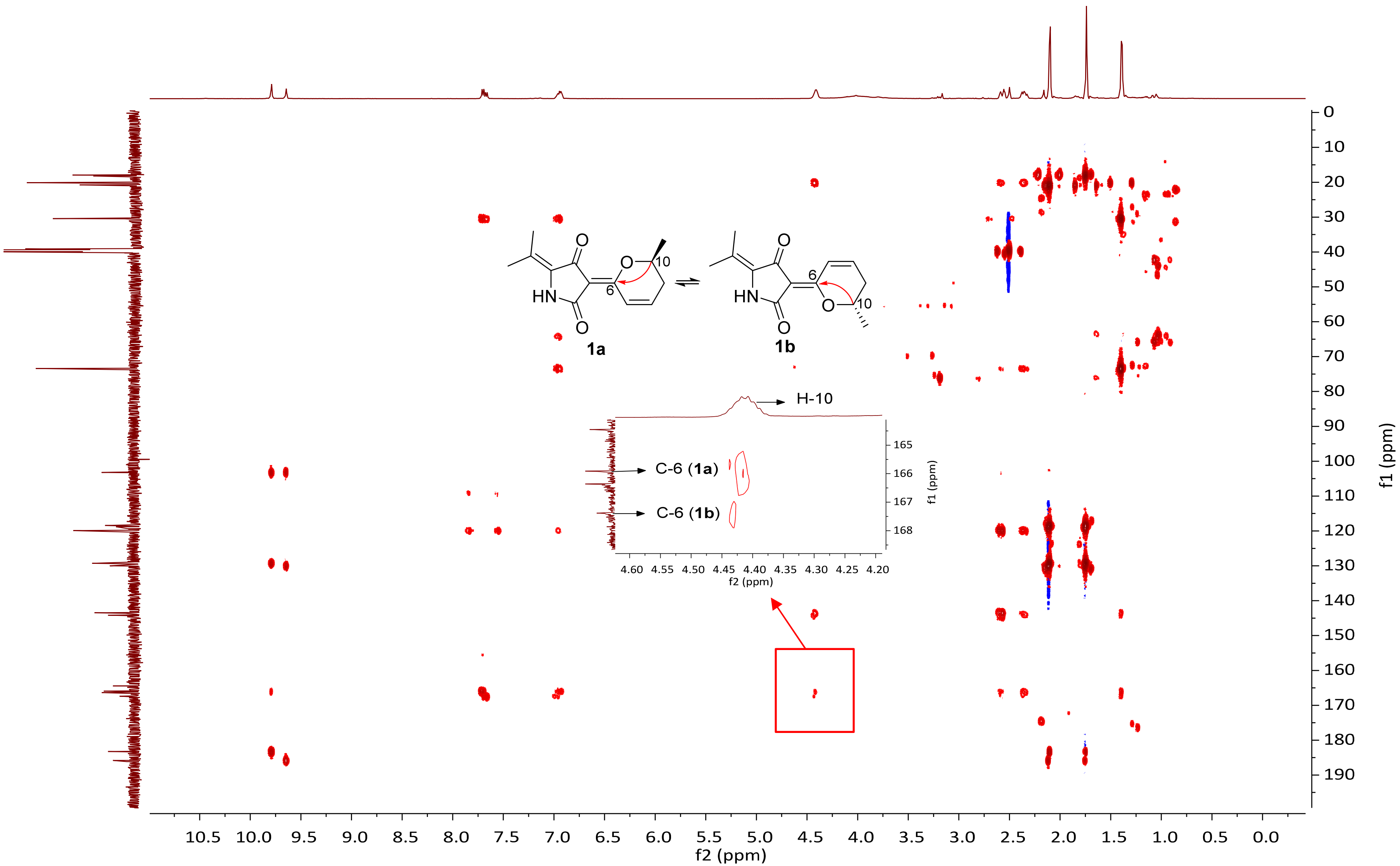

Figure S8. Positive ESIMS spectrum of cladodionen (**1**)

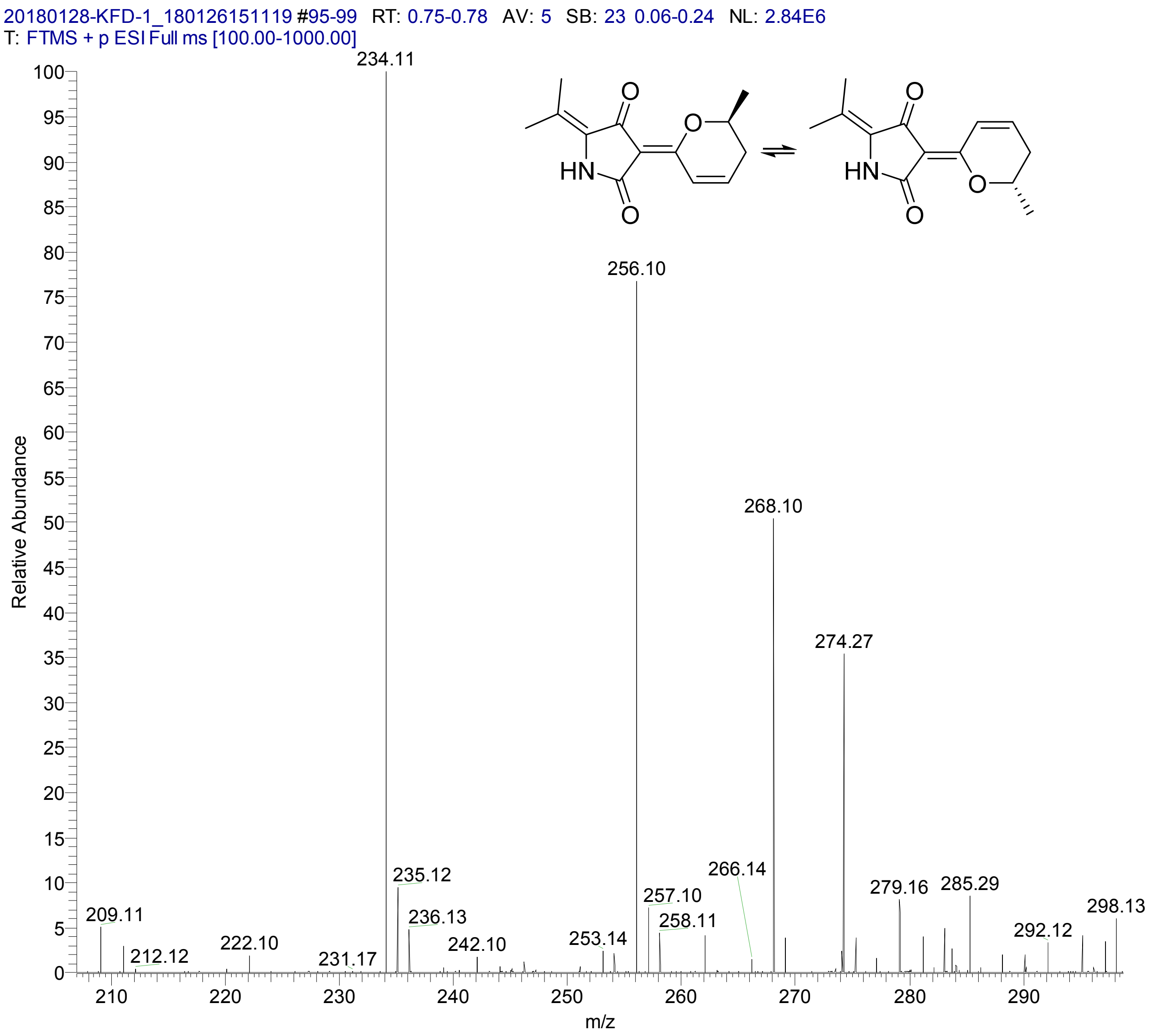

17 **Figure S9**. Positive HRESIMS spectrum of cladodionen (**1**)

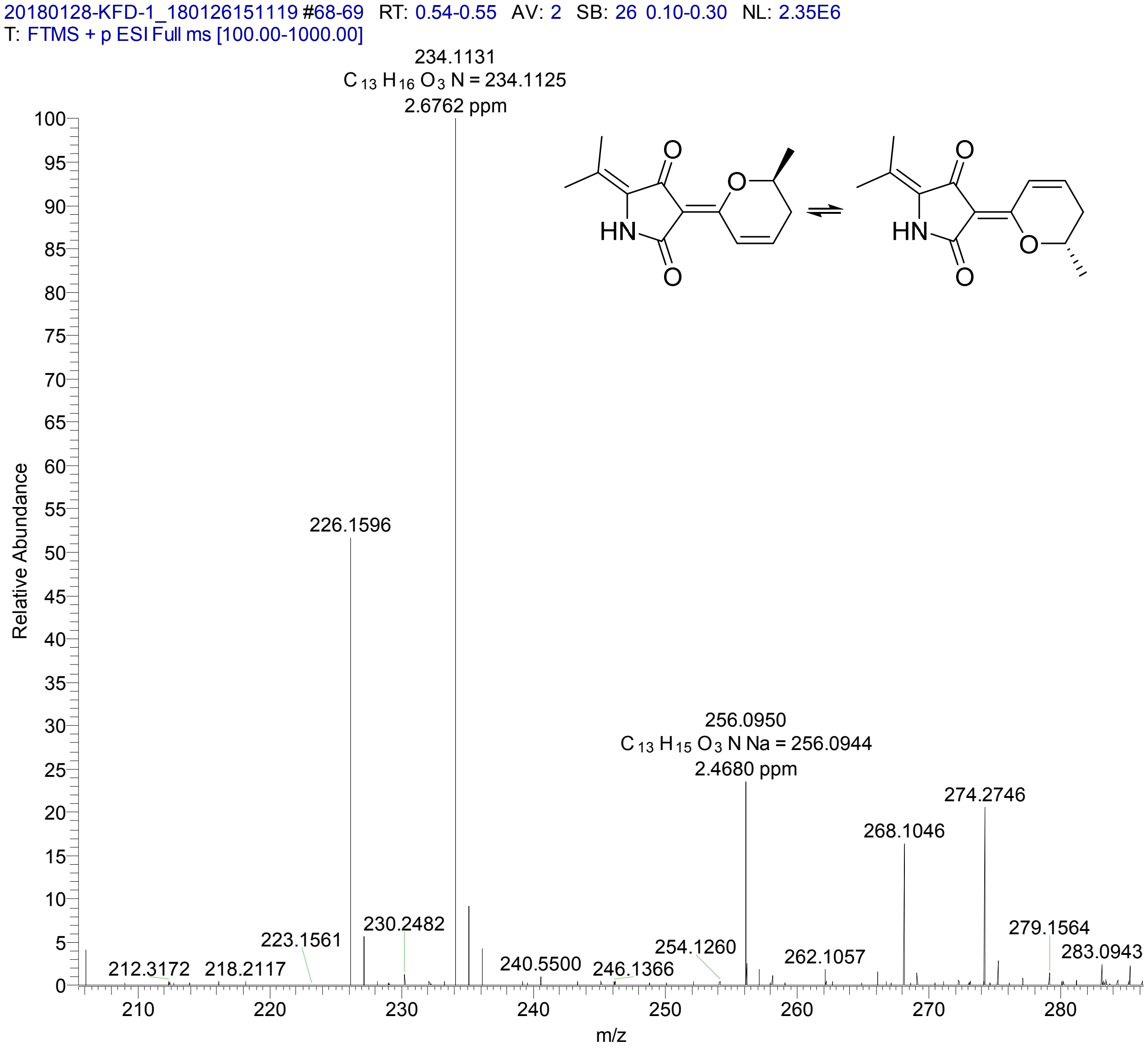

18

19

Figure S10. ^{1}H-NMR (500 MHz) spectrum of cladosacid (**2**) in $CDCl_3$

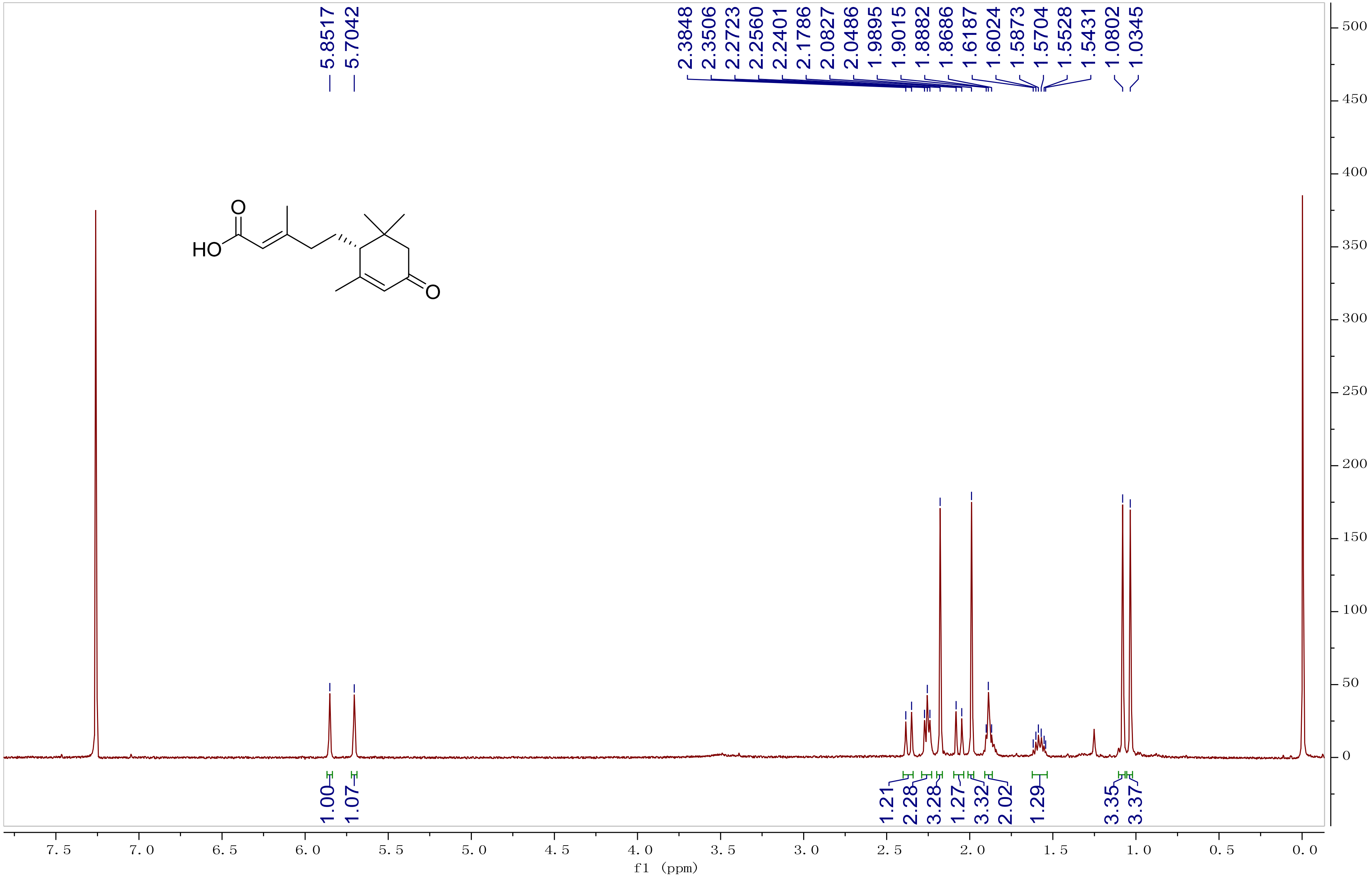

Figure S11. ^{13}C-DEPTQ (125 MHz) spectrum of cladosacid (**2**) in $CDCl_3$

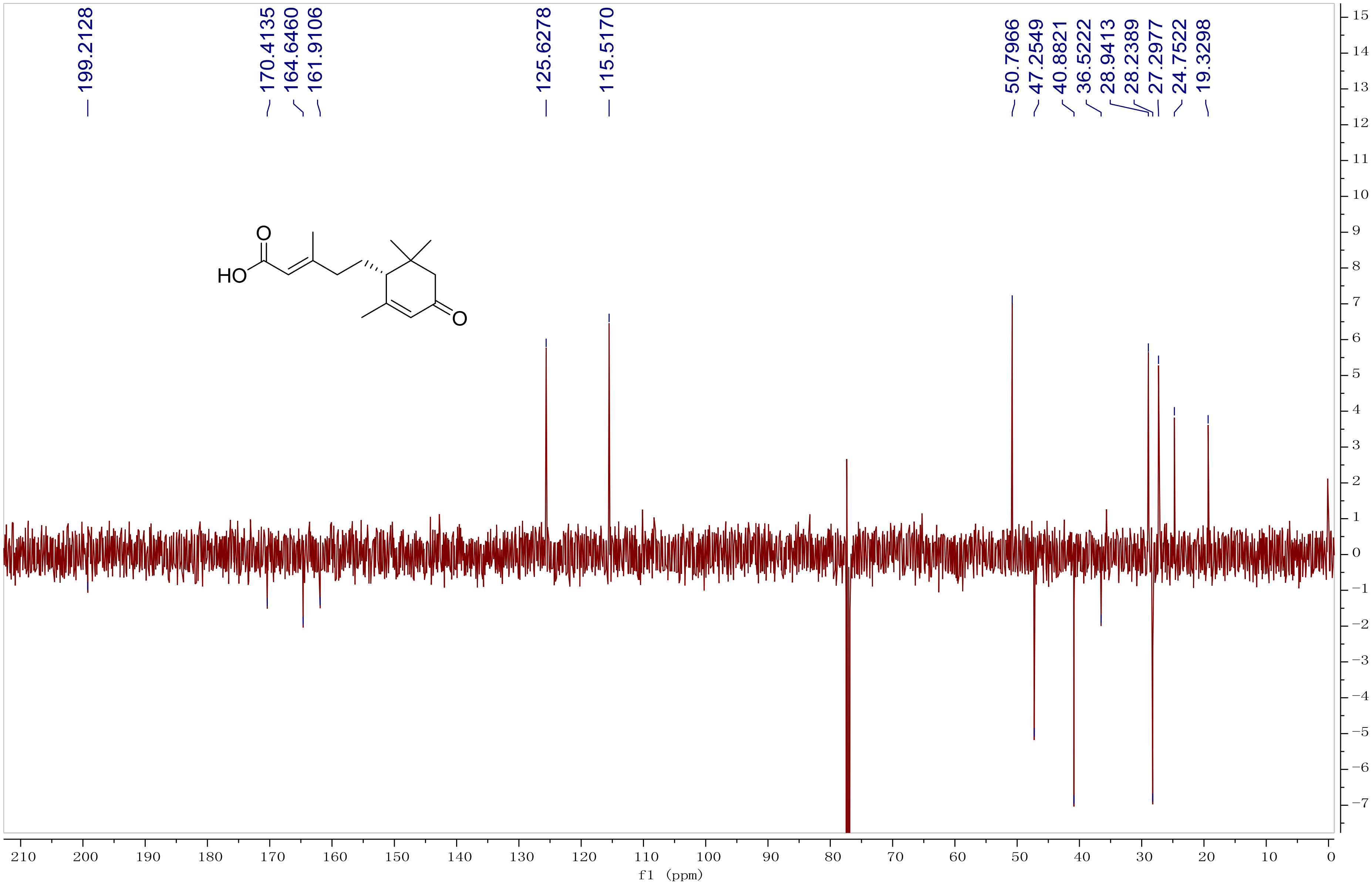

Figure S12. HSQC (500 MHz) spectrum of cladosacid (**2**) in $CDCl_3$

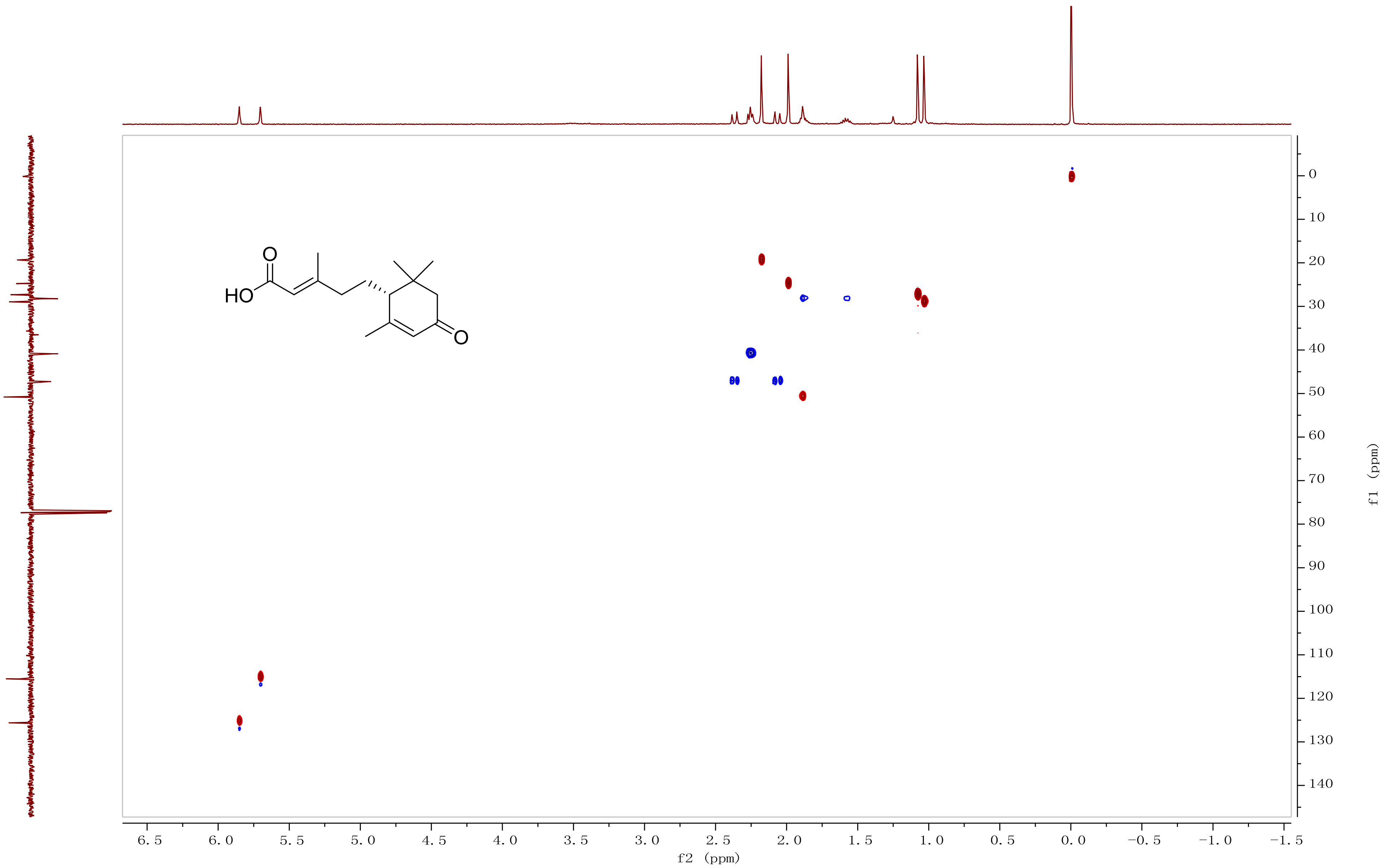

Figure S13. ^{1}H-^{1}H COSY (500 MHz) spectrum of cladosacid (**2**) in $CDCl_3$

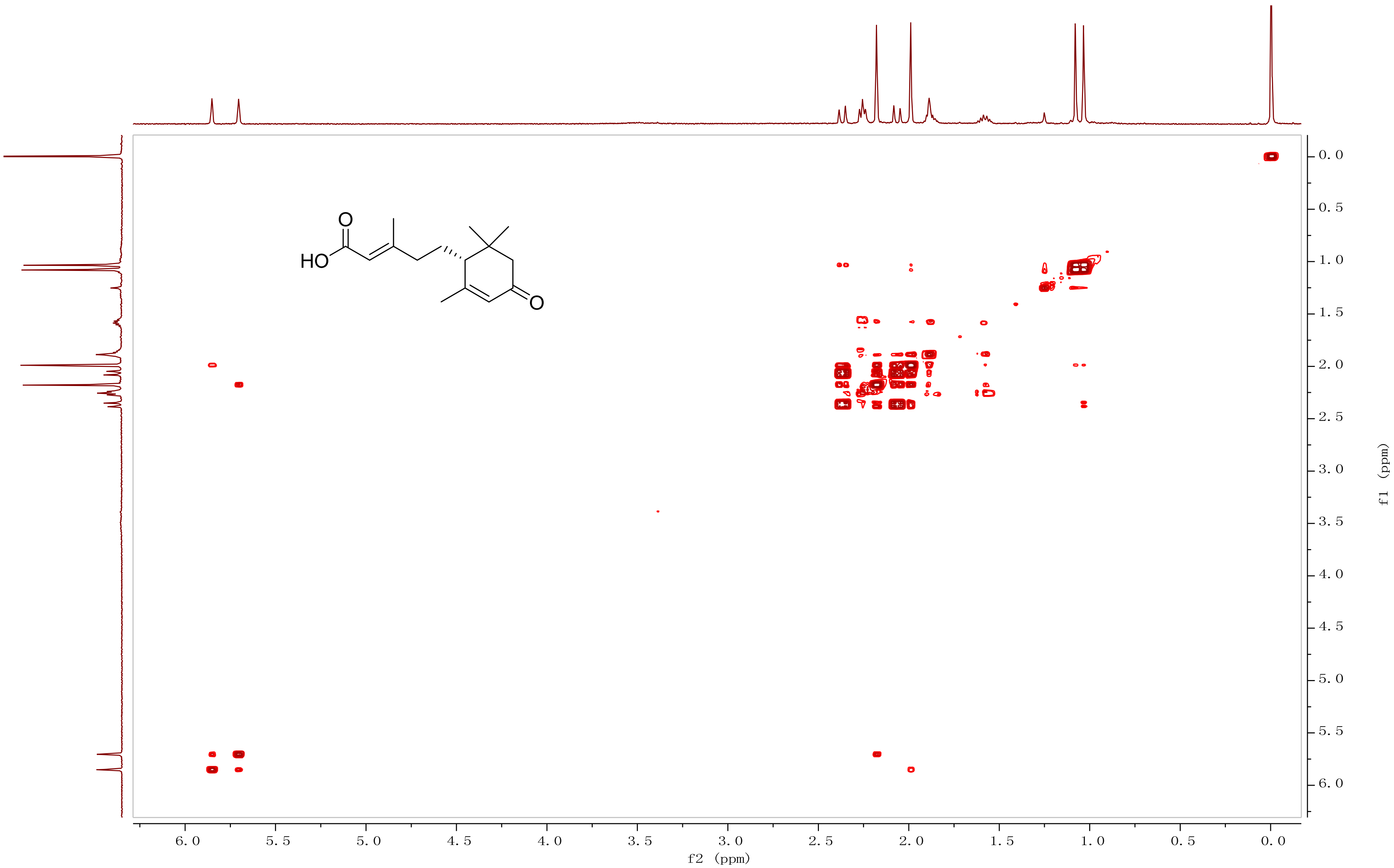

Figure S14. HMBC (500 MHz) spectrum of cladosacid (**2**) in $CDCl_3$

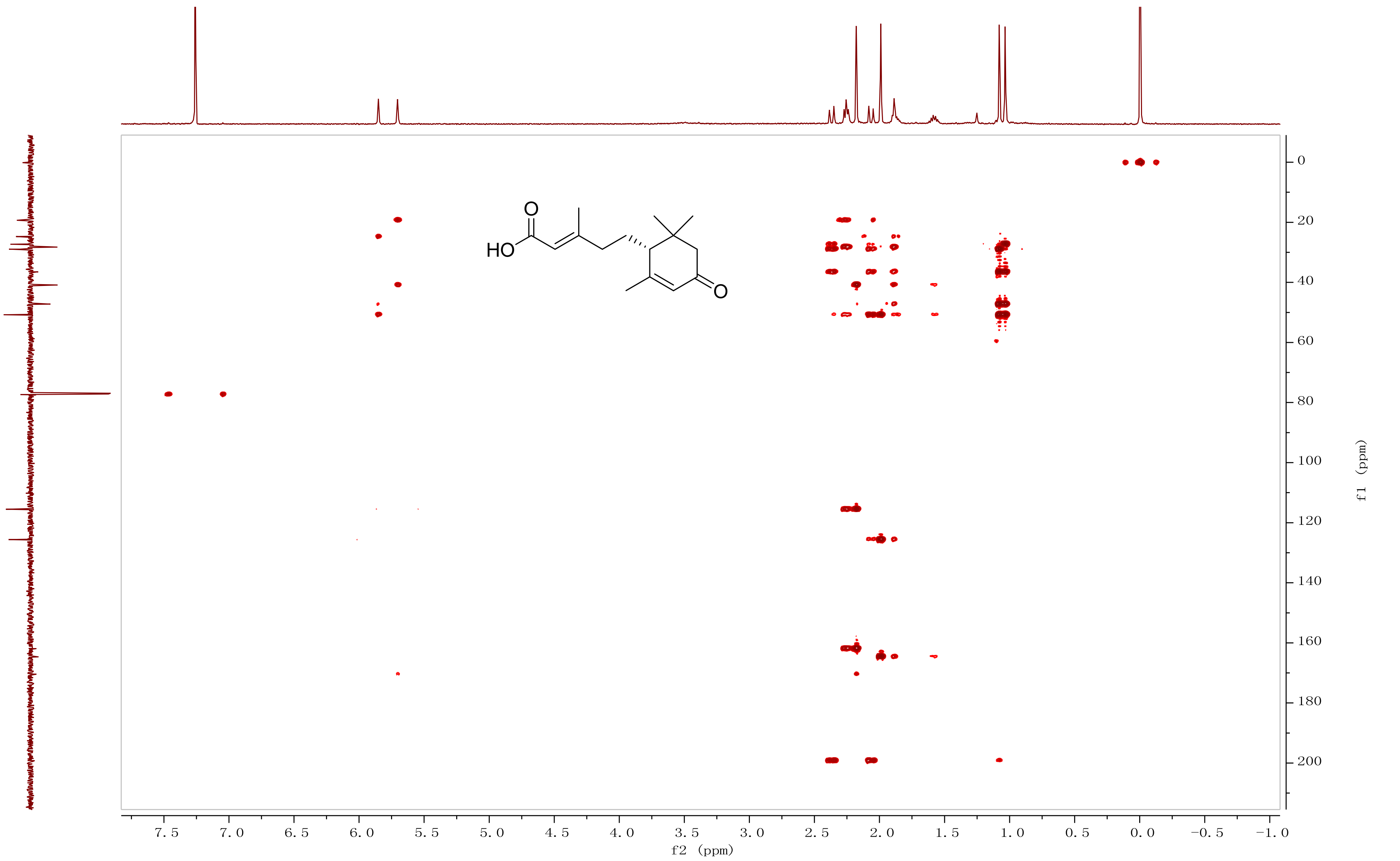

Figure S15. NOESY (500 MHz) spectrum of cladosacid (**2**) in $CDCl_3$

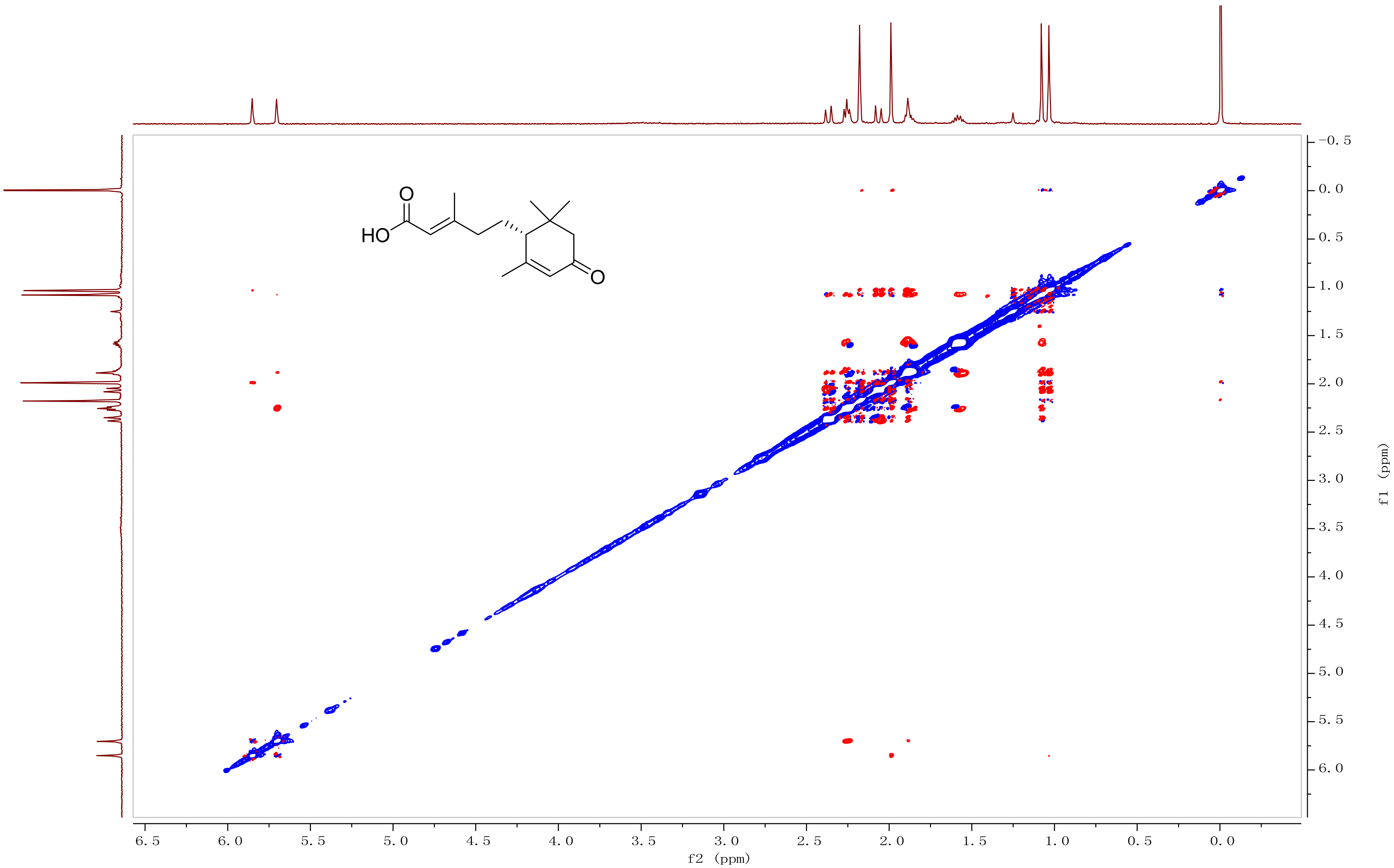

Figure S16. Negative ESIMS spectrum of cladosacid (**2**)

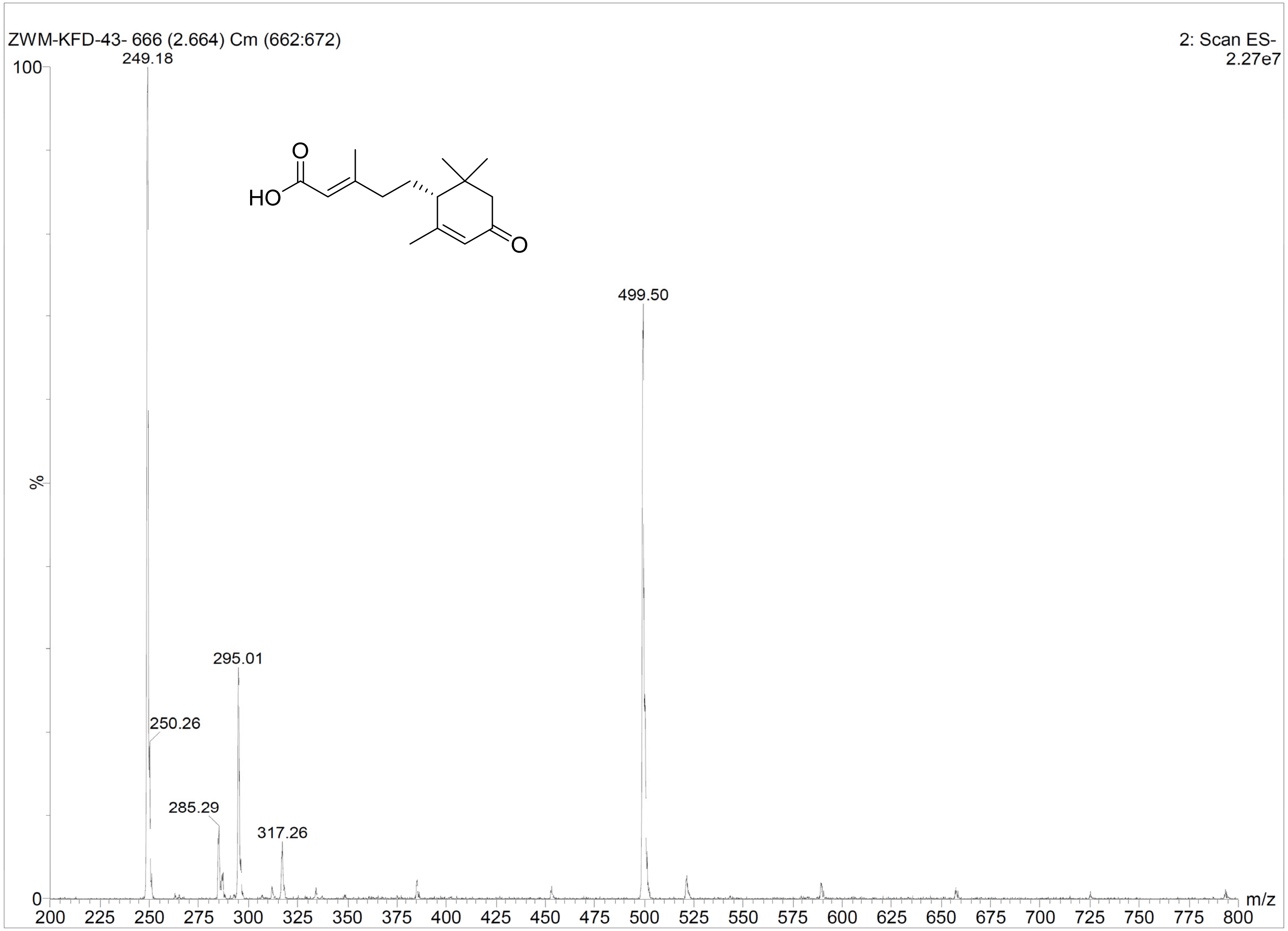

Figure S17. Negative HRESIMS spectrum of cladosacid (**2**)

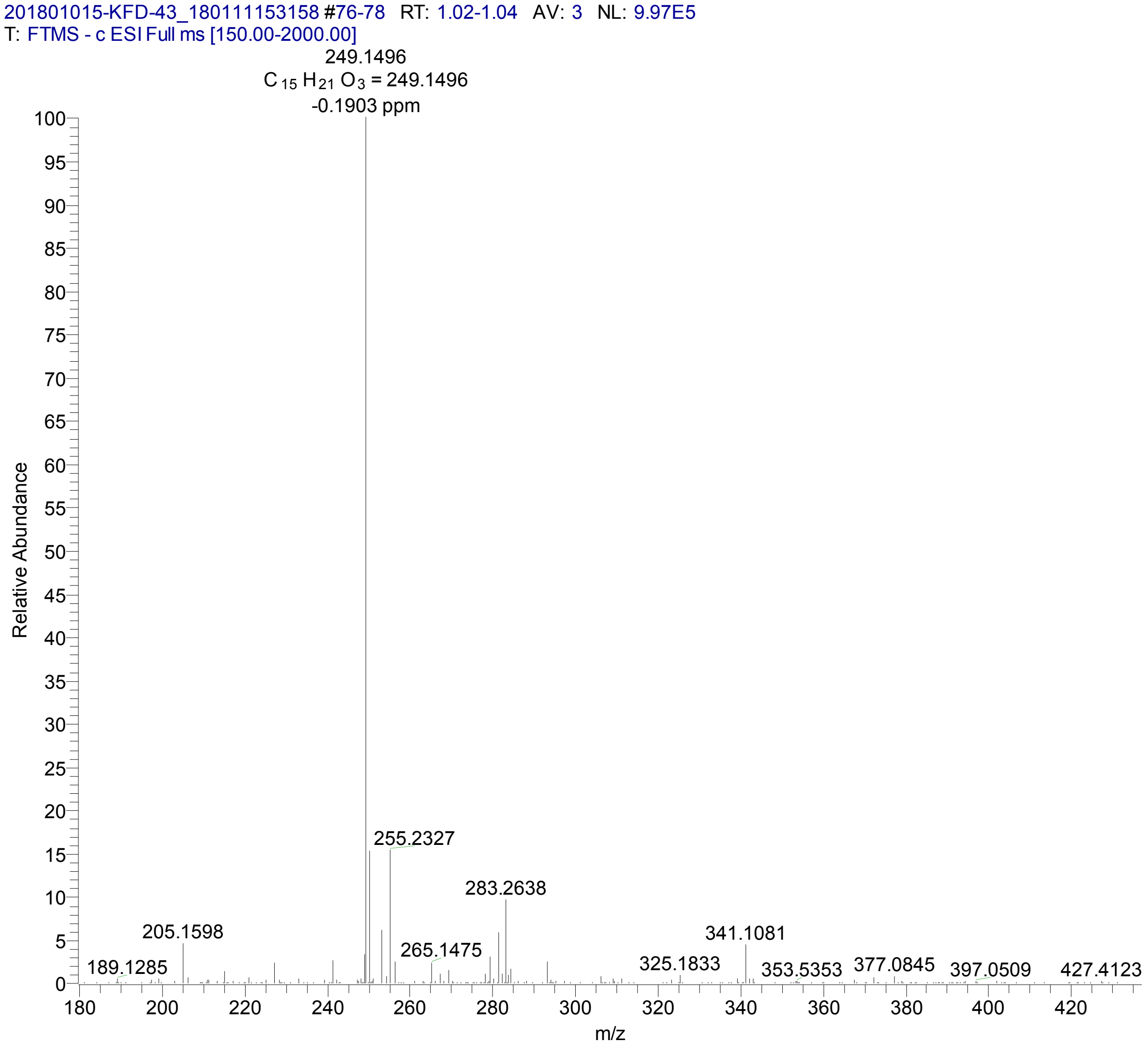

Figure S18. Positive HRESIMS spectrum of cladosacid (**2**)

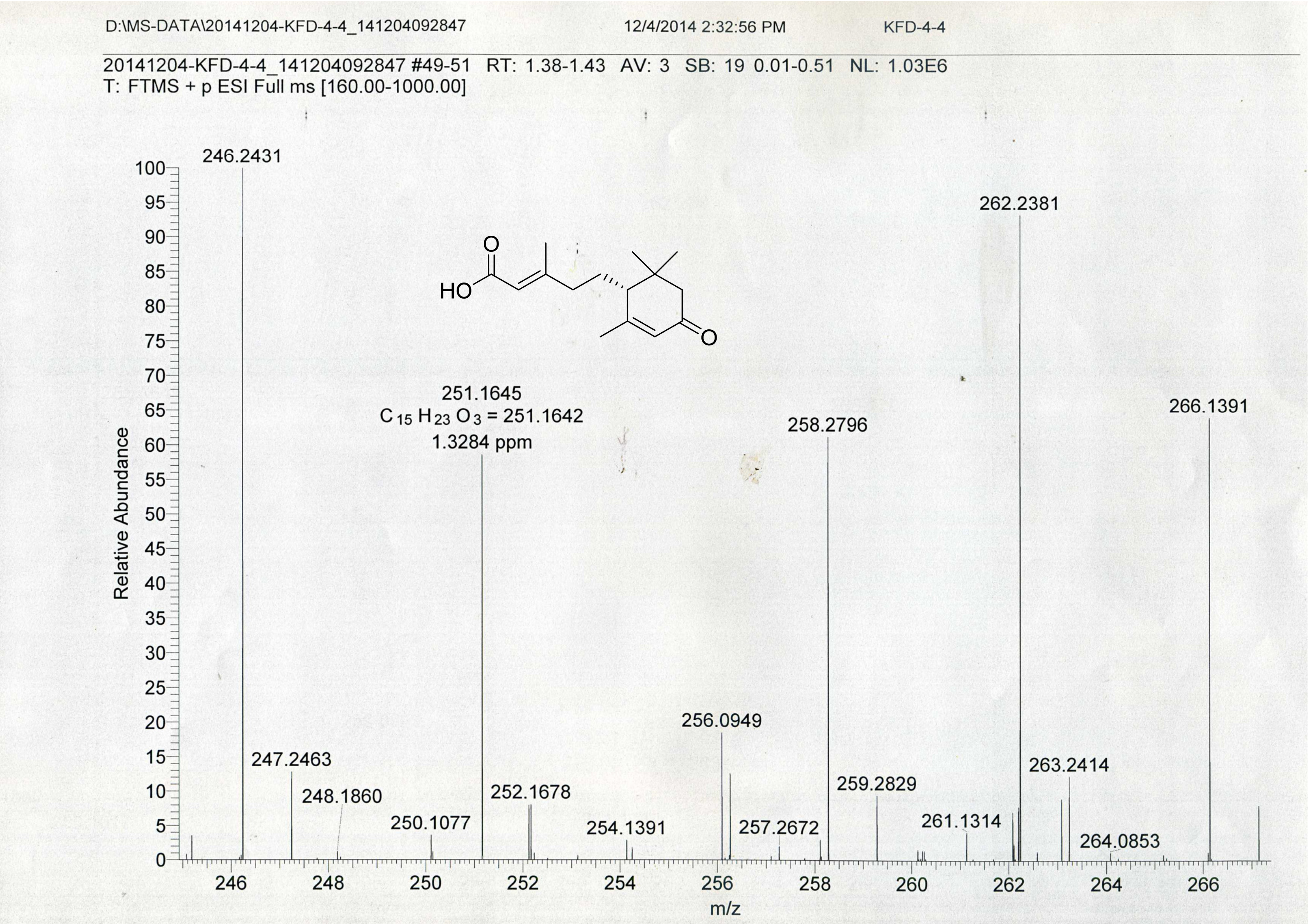

Figure S19. ^{1}H-NMR (500 MHz) spectrum of compound **3** in DMSO-d_6

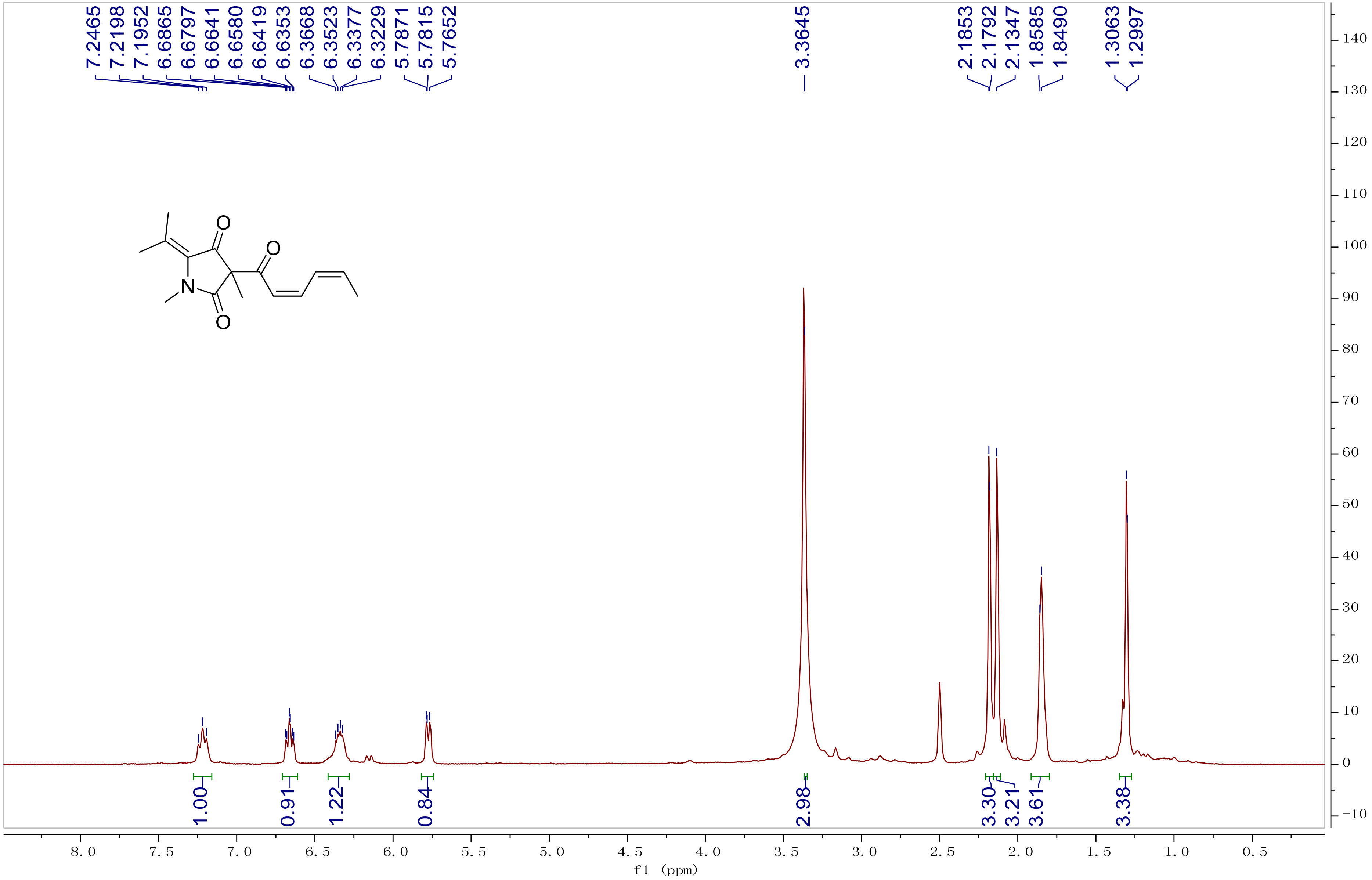

Figure S20. ^{13}C-NMR (125 MHz) spectrum of compound **3** in DMSO-d_6

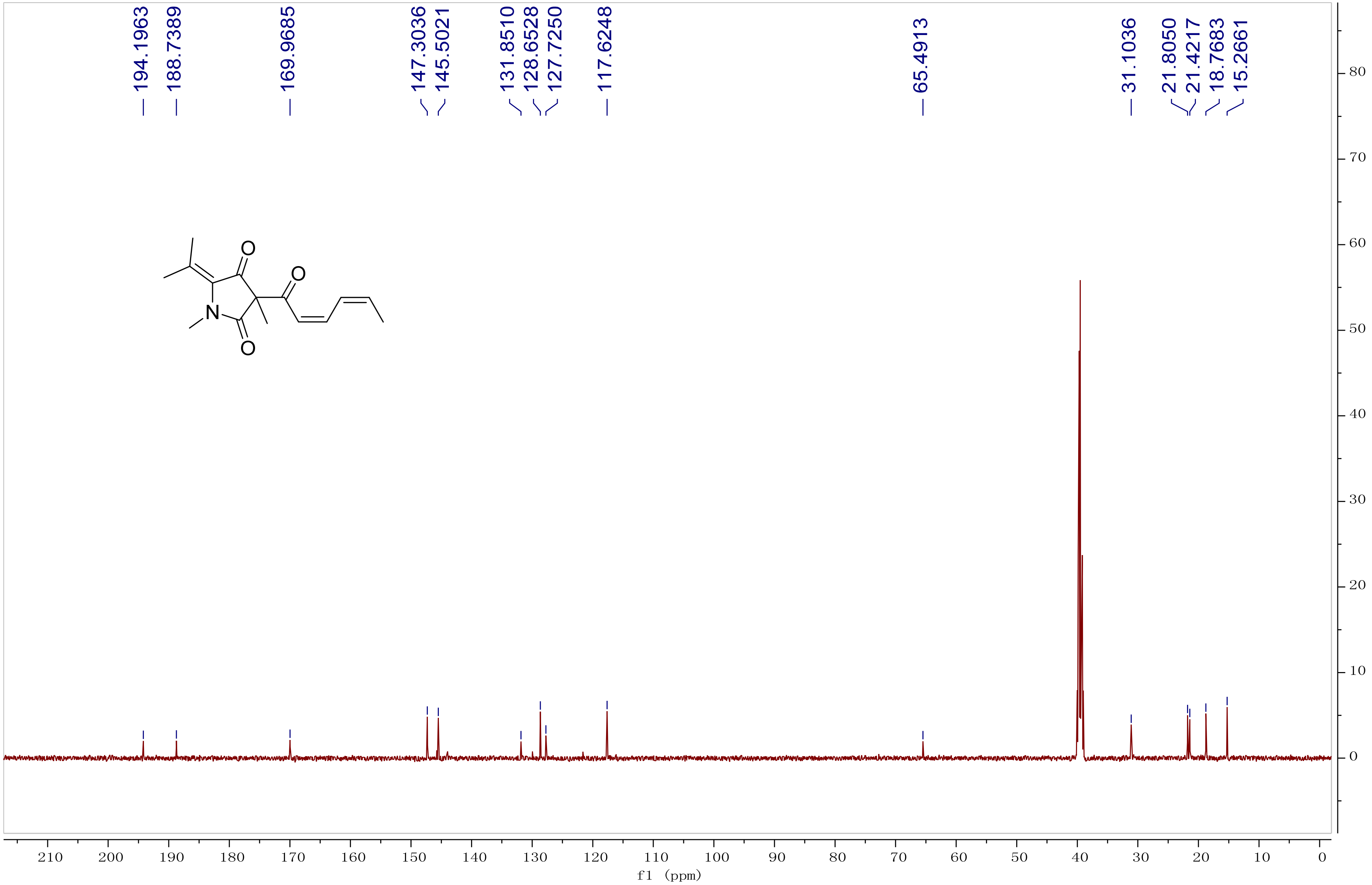

Figure S21. DEPT (125 MHz) spectrum of compound **3** in DMSO-d_6

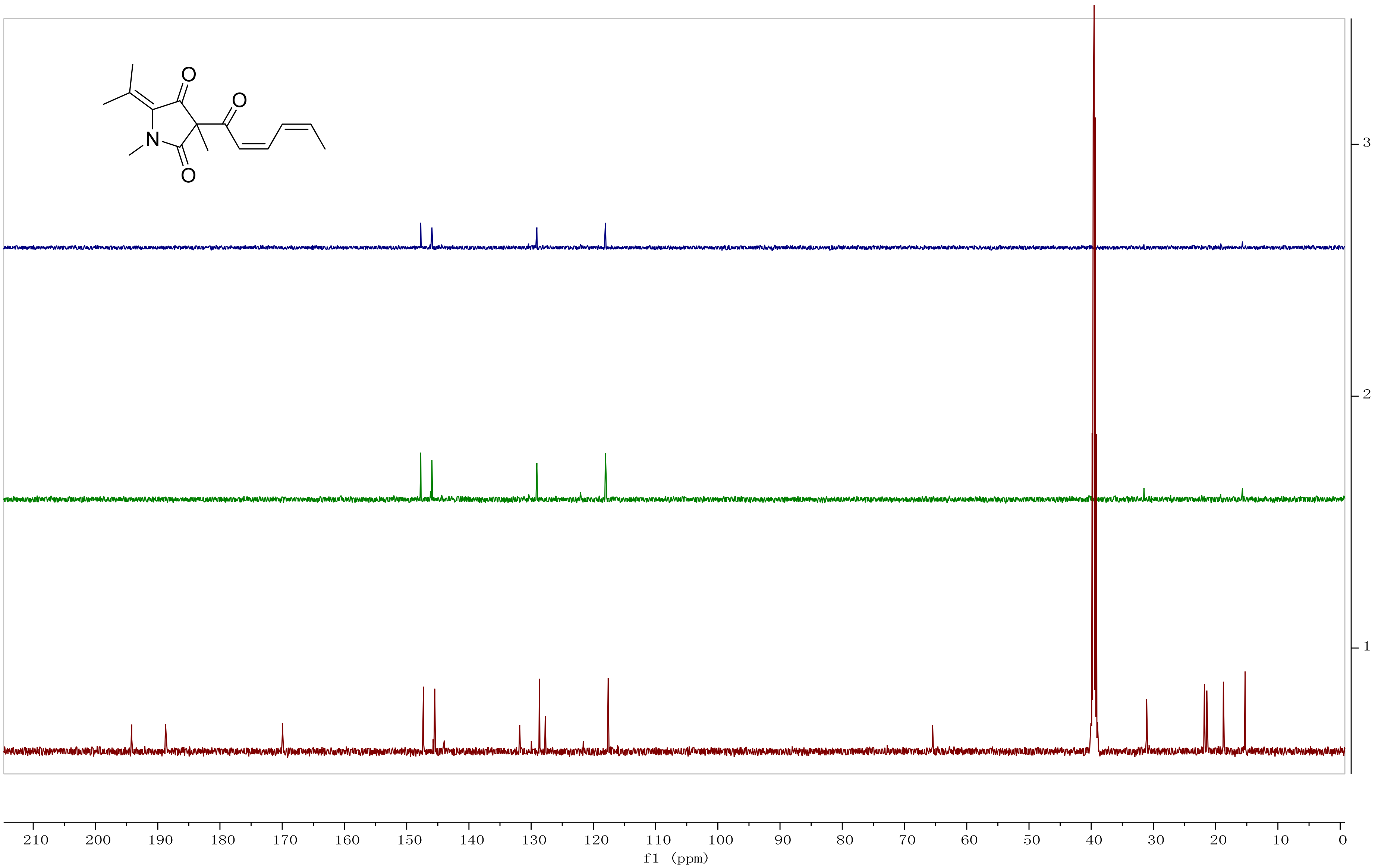

Figure S22. HMQC (500 MHz) spectrum of compound **3** in DMSO-d_6

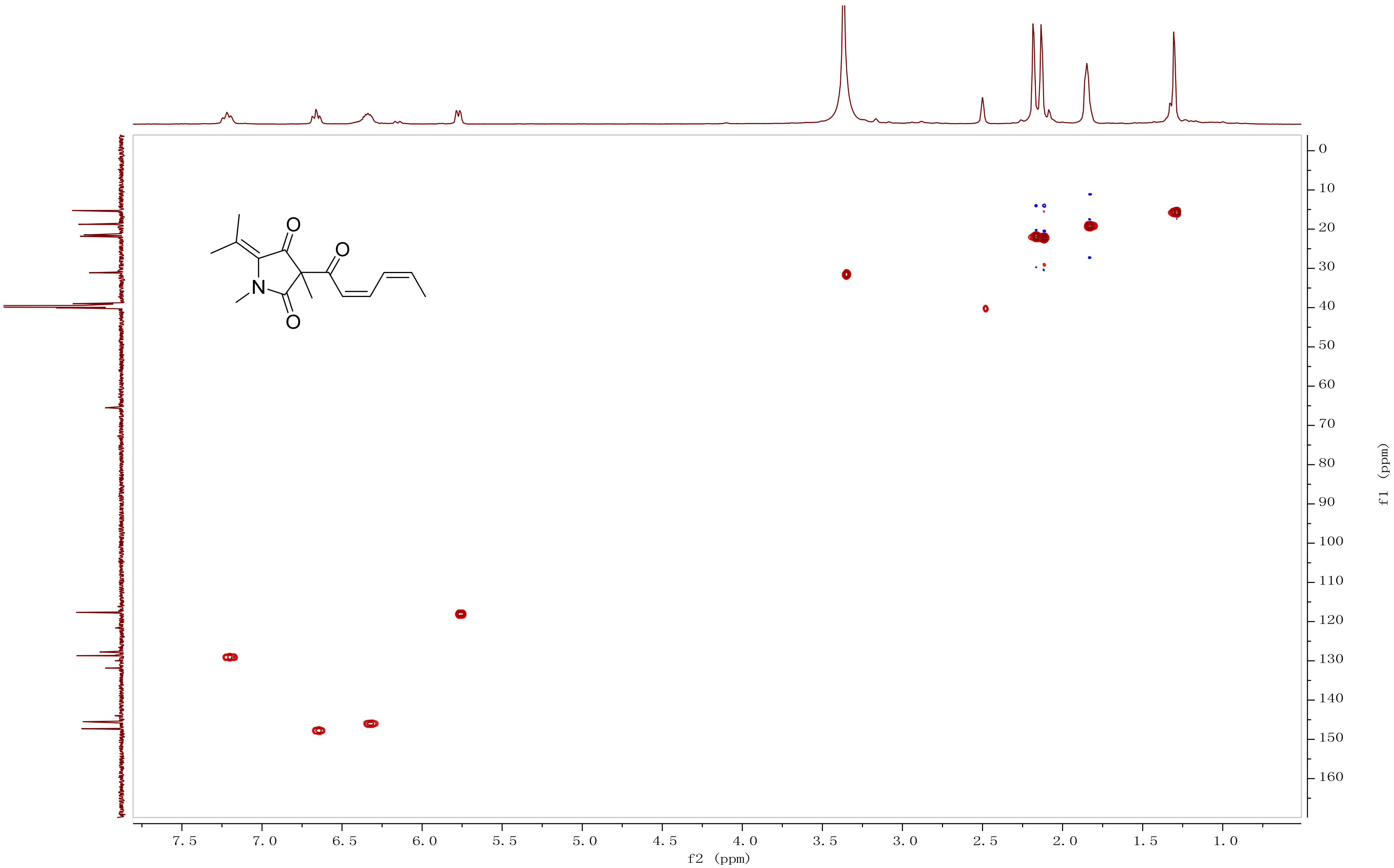

Figure S23. ^{1}H-^{1}H COSY (500 MHz) spectrum of compound **3** in DMSO-d_6

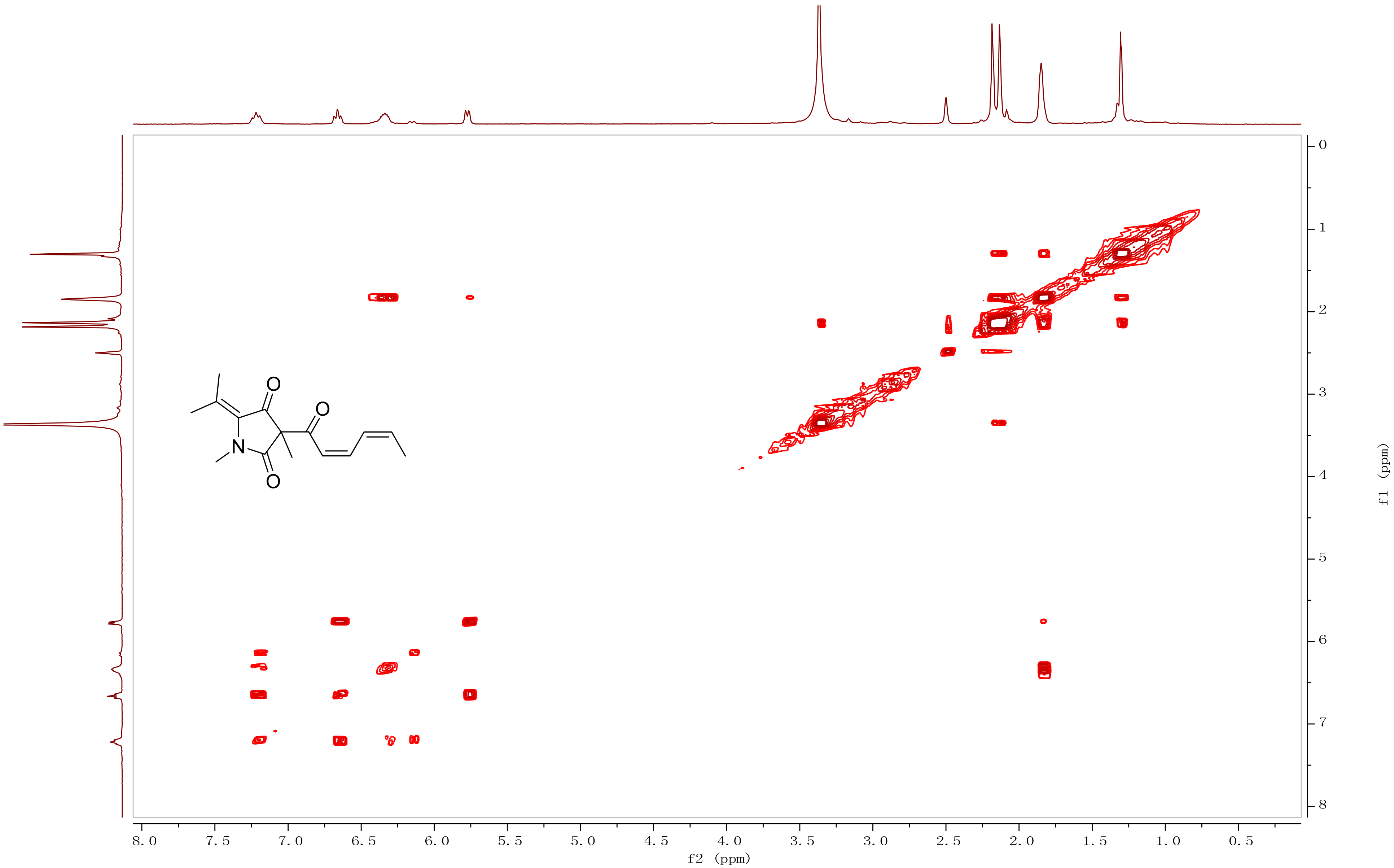

Figure S24. HMBC (500 MHz) spectrum of compound **3** in DMSO-d_6

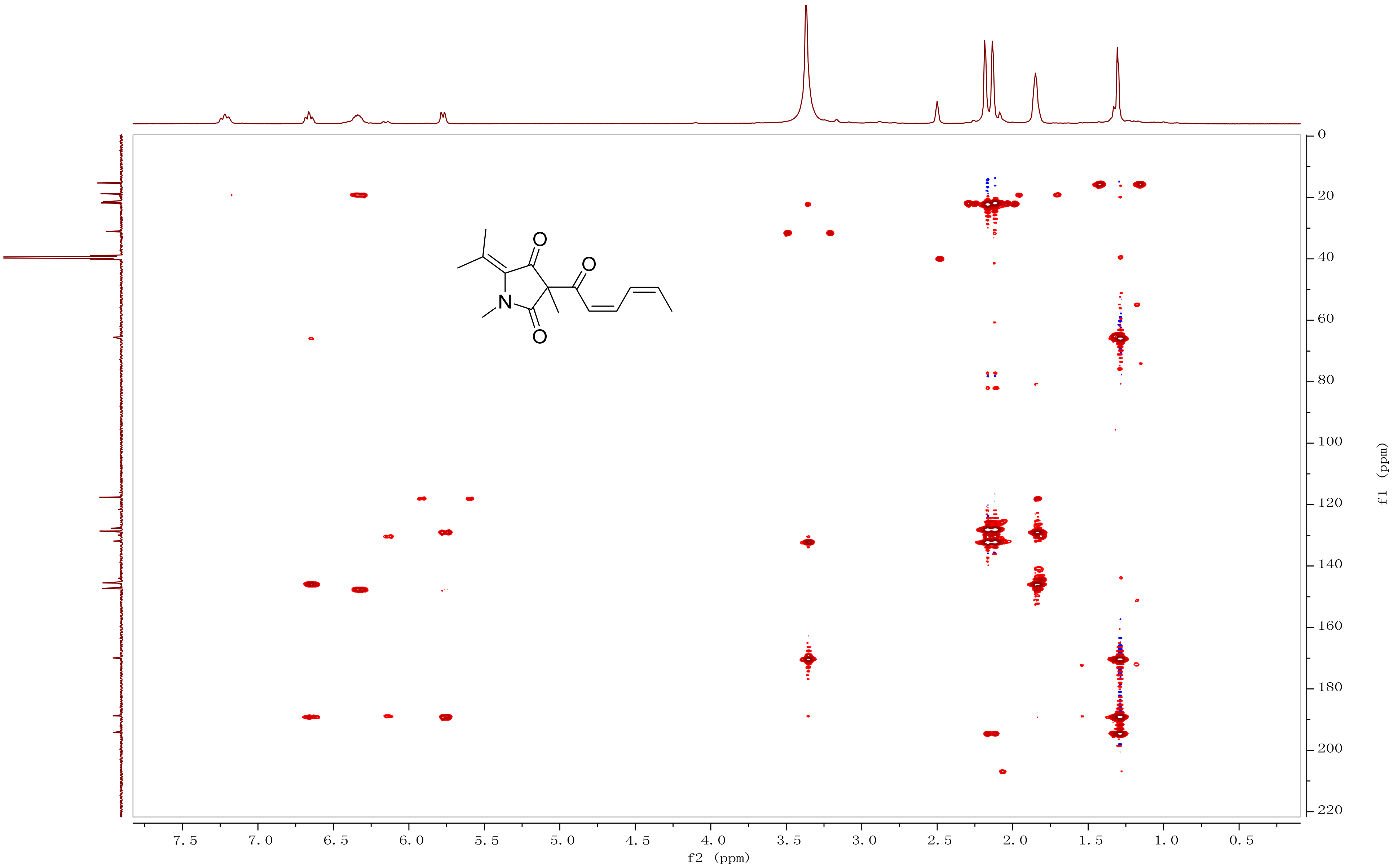

Figure S25. Positive ESIMS spectrum of compound **3**

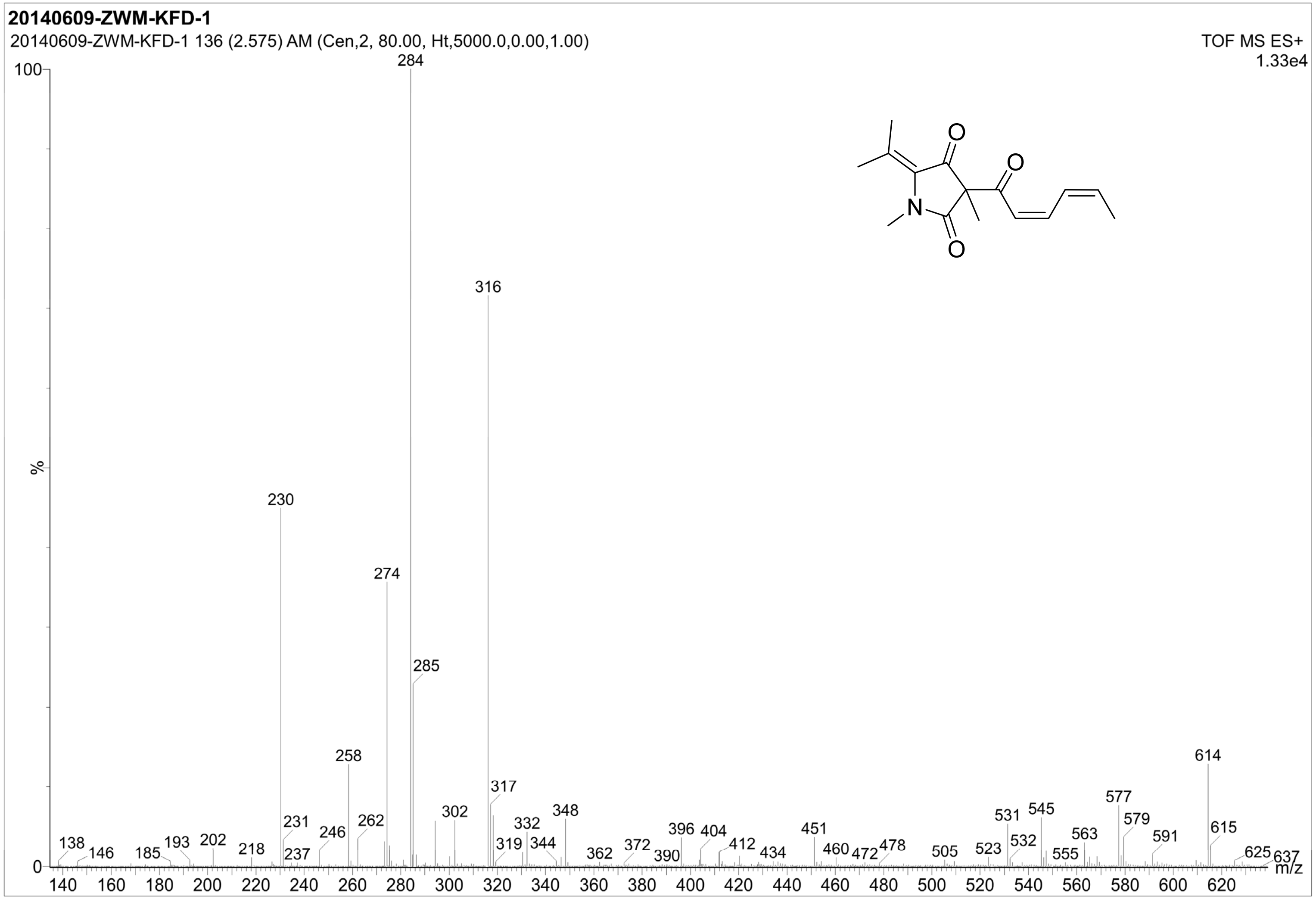

Figure S26. The ecological picture of the sponge sample

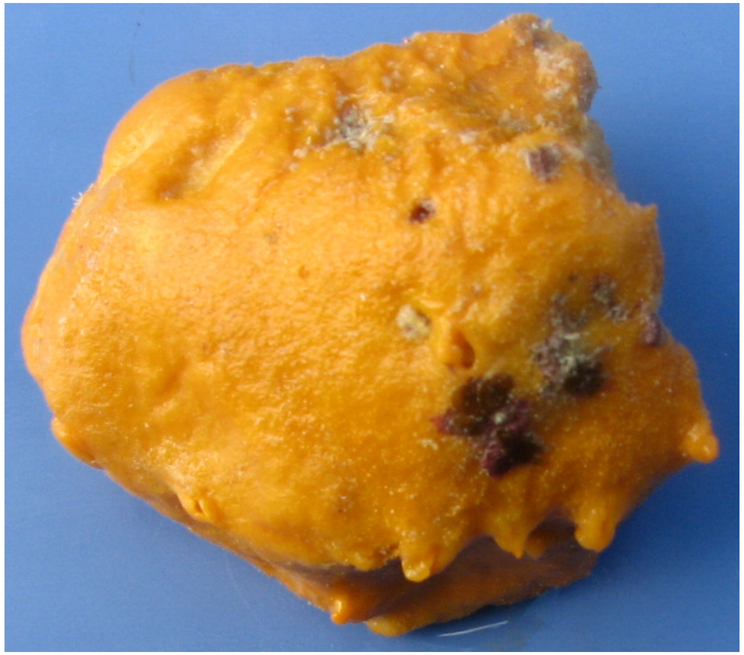

Supplement: Supplementary file 1 [file marinedrugs-16-00071-s001.pdf]
